# MicroRNA files in the prevention of intestinal ischemia/reperfusion injury by hydrogen rich saline

**DOI:** 10.1042/BSR20191043

**Published:** 2020-01-24

**Authors:** Weifeng Yao, Xiaoyu Lin, Xue Han, Lanfen Zeng, Anshun Guo, Yu Guan, Ziqing Hei, Jianpei Liu, Pinjie Huang

**Affiliations:** 1 Department of Anesthesiology, The Third Affiliated Hospital of Sun Yat-sen University, Guangzhou 510000, China; 2 Department of Anesthesiology, Sun Yat-sen Memorial Hospital, Sun Yat-sen University, Guangzhou 510000, China; 3 Department of Anesthesiology, Affiliated Hospital of Guangdong Medical University, Zhanjiang, Guangdong, China; 4 Department of Gastrointestinal Surgery, The Third Affiliated Hospital of Sun Yat-sen University, Guangzhou, China

**Keywords:** Inflammation, Intestinal Ischemia/Reperfusion Injury, Oxidative stress

## Abstract

Background: Hydrogen-rich saline (HRS) has been proven effective against ischemia/reperfusion (I/R) injury. However, knowledge on the underlying signaling events remain poor. Having recent highlight of microRNAs (miRNAs) in mediating intestinal I/R injury, we hypothesized that HRS may protect intestine against I/R injury by regulating miRNAs.

Method: Mice were given intraperitoneal injection of saline or HRS once daily for five consecutive days before undergoing intestinal I/R that was induced by 60-min ischemia followed by 180-min reperfusion of superior mesenteric artery. The intestine was collected for histopathological assay, miRNA microarray profiling, Real-Time PCR, and Western blotting. Next, miR-199a-3p mimics or inhibitors were transfected into IEC-6 cells to explore the relationship between HRS treatment and miR-199a-3p.

Results: I/R-induced mucosal injury and epithelial cells apoptosis were attenuated by HRS pretreatment. A total of 64 intestinal I/R-responsive miRNAs were altered significantly by HRS pretreatment, in which we validated four novel miRNAs with top significance by Real-Time PCR, namely miR-199a-3p, miR-296-5p, miR-5126, and miR-6538. Particularly, miR-199a-3p was drastically increased by I/R but reduced by HRS. Computational analysis predicts insulin-like growth factor (IGF)-1, mammalian target of rapamycin (mTOR), and phosphoinositide-3-kinase (PI3K) regulatory subunit 1 as targets of miR-199a-3p, suggesting involvement of the pro-survival pathway, IGF- 1/PI3K/Akt/mTOR. In *in vitro* experiment, HRS treatment reduced miR-199a-3p level, increase IGF-1, PI3K and mTOR mRNA expression, restore IEC-6 cells viability, and this protective effects were reversed under miR-199a-3p mimics treatment.

Conclusion: Collectively, miR-199a-3p may serve a key role in the anti-apoptotic mechanism of HRS that contributes to its protection of the intestine against I/R injury.

## Introduction

Ischemia/reperfusion (I/R) injury occurs in a tissue that undergoes a period of blood flow restriction followed by restoration [1]. The intestine is one of the organs that is most susceptible to I/R injury, which is problematical in surgical procedures requiring thoracic or abdominal vascular occlusion such as cardiopulmonary bypass and intestinal transplantation. It can also take place in hemorrhagic or septic shock [2]. The devastating sequelae of intestinal I/R injury includes deteriorated integrity and motility of mucosa, bacterial translocation to the circulation, and overproduction of reactive oxygen species (ROS) [2,3]. These events are strong drive of systemic inflammation and multi-organ failure, imposing high morbidity and mortality in patients subjected to intestinal I/R [4].

Saline saturated with hydrogen, a proven antioxidant, has been shown to protect tissue by antioxidation and anti-inflammation in clinic [5], as well as in various animal models of I/R [6,7], including intestinal I/R [8]. Such hydrogen-rich saline (HRS), by selectively reacting with hydroxyl radicals and peroxynitrite, the most toxics of ROS, can effectively reduce cellular oxidative stress and apoptosis [6,9]. Administration of HRS prior to intestinal I/R has been shown to suppress lipid oxidation, proinflammatory cytokines, and crypt cell apoptosis in intestinal mucosa, conferring significant protection of the intestine [3,8]. However, besides these antioxidant qualities, much less is known on the role of HRS in cell signaling.

In recent years, accumulating research has highlighted the functional significance of microRNAs (miRNAs) in control of intestinal I/R injury [10,11]. MiRNAs are small noncoding RNAs that silence gene expression during post-transcription [12]. By targeting miRNAs related to intestinal I/R injury, such as miR-665 and miR-381, studies have demonstrated histological and functional improvements in the intestine following I/R [13,14]. Therefore, we infer that HRS may protect intestine from I/R injury by regulating miRNAs. However, no literature has reported miRNAs expression in HRS-treated intestinal I/R. The present study aims to identify novel miRNAs involved in the protective mechanism of HRS in intestinal I/R injury.

## Materials and methods animals

Thirty-two male C57BL/6 mice (age 7–8 weeks and body weight 20–24 g) were used in the present study. Mice were housed in a room illuminated 12-h light–dark cycle. Mice were allowed access to water and food *ad libitum*. All procedures were approved by the Institutional Animal Care and Use Committee at The Third Affiliated Hospital of Sun Yat-Sen University and performed in accordance with the National Institutes of Health guidelines for the use of experimental animals. The committee is guided by the Care and Use of Laboratory Animals (1996). All of the animal experiments in the present study were carried out in experimental animal center of Sun Yat-sen University, which were approved by Animal Ethics Committee of Sun Yat-sen University (SYSU-IACUC-2017-B432).

### Experiment design

Thirty-two mice were randomly divided into four groups (*n* = 8) as the following:

- Sham group, mice were given daily intraperitoneal injection (i.p.) of saline (the same volume as HRS) for five consecutive days before sham operation.
- I/R group, same administration scheme as sham group before intestinal I/R operation.
- HRS group, daily i.p. injection of HRS (3 μmol/kg) for five consecutive days before sham operation.
- HRS+I/R group, same administration scheme as HRS group before intestinal I/R operation.

All the mice were killed by high concentration of carbon dioxide. Intestine tissue at 10 cm to the terminal ileum (0.5 cm) was collected and fixed in 10% formaldehyde, followed by embedding in paraffin. Next, the whole small intestine was removed and washed thoroughly with saline (0°C). Intestinal epithelium was exposed by a longitudinal cut, and was rinsed completely with saline and stored at −80°C freezer.

### Hydrogen-rich saline preparation

To obtain HRS, hydrogen was dissolved in saline (0.9%) for 6 h under pressure of 0.4 MPa to a supersaturated level using a hydrogen rich saline-producing apparatus (ZhongKeHuiHeng, Beijing, China). The saturated hydrogen saline was stored fully in an aluminum bottle at 4°C under atmospheric pressure and sterilized by gamma radiation and freshly prepared to maintain the saturated concentration at 0.6 mmol/l. The content of hydrogen in HRS was measured by a hydrogen gas concentration measurement instrument (TRUSTLEX ENH-1000, Japan).

### Intestinal ischemia/reperfusion model

Intestinal I/R was performed as previously described [15]. Briefly, surgical area of mouse abdomen was sterilized and opened with middle abdominal incisions under anesthesia (ketamine and chlorpromazine). Intestinal I/R were carried out by clipping of the superior mesenteric artery (SMA) completely with a microvascular clamp. After 60 min of occlusion, intestinal blood flow was restored by removing the microvascular clamp. This I/R regimen (60-min ischemia followed by 180-min reperfusion) was chosen because this time course provoked the most severe intestinal inflammation and oxidative stress in our previous study [16]. Sham-operated mice were subjected to identical surgical interventions including laparotomy and vascular microdissection for the same operation period without SMA occlusion.

### Intestinal cell hypoxia/reoxygenation

Intestinal cells line IEC-6 was obtained from ATCC (Manassas, VA, U.S.A.). Hypoxia/Reoxygenation (HR) was carried out as described in our previous studies [17]. In brief, cells were cultured under hypoxia gas mixture (5% CO_2_, 94% N_2_ and 1% O_2_, 37°C) in a hypoxia incubator (Eppendorf Company, Hamburg, Germany) for 2 h and after hypoxia, cells were then placed in 5% CO_2_ incubator for reoxygenation for 60 min. The viability of IEC-6 was detected using the Cell Counting Kit-8 (CCK-8) method according to the manufacturer’s instructions (Roche, U.S.A.).

### IEC-6 cells transfection

IEC-6 cells (1–5 × 10^5^) were seeded into 24-well plates with 30–50% cell confluence. Negative control, inhibitors or mimics of miR-199a-3p (RiboBio, China) were diluted with riboFECT™ CP buffer (RiboBio, China), respectively. Mix diluted Negative control, inhibitors or mimics of miR-199a-3p with riboFECT™ CP Reagent Transfection Agent (RiboBio, China) and then incubate the mixture for 10 min at room temperature. Add the mixture to the cell culture medium and mix gently. Negative control, inhibitors or mimics of miR-199a-3p were diluted at a final concentration of 50 nM. The transfected IEC-6 cells were cultured in 37°C for 48 h before hypoxia/reoxygenation or HRS treatment.

### Hydrogen-rich saline medium preparation

To obtain HRS medium, hydrogen was dissolved in Dulbecco’s modified eagle medium (DMEM) for 6 h under pressure of 0.4 MPa to a supersaturated level. The saturated HRS medium was stored fully in an aluminum bottle at 4°C under atmospheric pressure and freshly prepared to maintain the saturated concentration at 0.6 mmol/l. IEC-6 cells were pretreatment with HRS medium for 24 h before cells subjected to hypoxia/reoxygenation.

### Evaluation of histological injury

Sections of 5-μm thickness from intestinal paraffin block were prepared. Tissue section was then stained with hematoxylin–eosin (H&E) after dewaxing hydration. Five randomly selected fields (×200) were captured from each slide using a light microscope in a blinded manner. The severity of intestinal injury was graded by two histopathologists who were initially blind to the experiment based on the criteria of *Chiu’s* method [18,19].

### TUNEL assay

Intestinal paraffin block sections (5 μm) were prepared for terminal deoxynucleotidyl transferase (TdT)-mediated dUTP nick end labeling (TUNEL) detection. TUNEL assay was performed according to the manufacturer’s instructions (Roche, U.S.A.). Five randomly selected fields (×200) were captured from each slide using a light microscope in a blinded manner. TUNEL positive cells were counted automatically by an imaging processing software (ImageJ, NIH, U.S.A.).

### RNA isolation

Total RNA was extracted using Trizol reagent (Invitrogen, California, U.S.A.) according to the protocol of the manufacturer. The quantity and purity of total RNA were monitored using NanoDrop ND-1000 spectral photometer. The integrity of the RNA was assessed with the RNA 6000 Nano Lab Chip Kit (Agilent, California, U.S.A.) in combination with the Bioanalyzer 2100 (Agilent Technologies, California, U.S.A.) with RIN number >8.0.

### Small RNA library preparation

An input of 1 μg of total RNA were used to prepare small RNA library according to manufacturer’s instructions of TruSeq Small RNA Sample Prep Kit (Illumina, SanDiego, U.S.A.). Briefly, RNA molecules were ligated to 5’ and 3’ adaptors successively and converted to cDNA by reverse transcription followed by PC amplification. PCR products were purifed using gel purifcation (6% Novex TBE gels). Quality control of each library sample was performed using the High Sensitivity DNA LabChip Kit (Agilent Technologies) on the 2100 Bioanalyzer (Agilent Technologies).

### Next-generation sequencing

The purified cDNA libraries were indexed with unique adapters and performed on an Illumina Hiseq 2000 at the BGI (Guangzhou, China) following the manufacturer’s instructions for instrument use. Illumina’s Sequencing Control Studio software version 2.8 (SCS v2.8) was used to obtain raw sequencing reads, which following real-time sequencing image analysis and base-calling by Illumina’s Real-Time Analysis version 1.8.70 (RTAv1.8.70).

### Quality control, mapping, and differential expression analysis

Following the removal of adapter sequences, low-quality reads, and common RNA families, only reads with the length between 15 and 55 base pairs were selected for further analyses. The reads were then mapped in miRBase 20.0 (http://www.mirbase.org/) by Bowtie [20]. Differential expression analyses including data quality assessment were performed with the DESeq software package based on the reads per sample.

### Real-time PCR (RT-PCR) assay

RT-PCR analyzed the expressions of mmu-miR-199a-3p, mmu-miR-296-5p, mmu-miR-5126, mmu-miR-6538, mmu-miR-342-3p, mmu-miR-677-5p, mmu-let-7c- 2-3p, and mmu-miR-574-5p in intestinal tissue and IGF1, PI3K, and mTOR in cultured cells. The total RNA was isolated from intestinal mucosa tissue using Trizol reagent (Gibco-BRL) according to the manufacturers’ instruction (Roche). Total RNA (5 μg) was reverse-transcribed into cDNA, and the PCR reaction mixtures were made by SYBR Green qPCR master mix (Toyoho Co., Ltd., Osaka, Japan). U6 snRNA was used as the internal control. Primer sequences were designed as follows: mmu-miR-199a-3p, ACAGUAGUCUGCACAUUGGUUA; mmu-miR-296-5p, AGGGCCCCCCCUC AAUCCUGU; mmu-miR-5126, GGGACCGGACGCCUCCUGCAGCUGCGGGA GCCCGUGGUUCCCCGGGCAACCGCGGGCGGGGCCGGGGGCGGGGCCCA; mmu-miR-6538, CCGUGCUGCCGGGCGGGGACCCCGCGGGCUCCGGGGCG GCGAUGGGGACUAUGCUCCGGGAUUCCGCUGGGUGACGGCCGCGUCCUC CUGGCCGCUUGCACUUGGGGAUC; mmu-miR-342-3p, UCUCACACAGAA AUCGCACCCGU; mmu-miR-677-5p, UUCAGUGAUGAUUAGCUUCUGA; mmu- let-7c-2-3p, UUCAGUGAUGAUUAGCUUCUGA; mmu-miR-574-5p, UGAGUGU GUGUGUGUGAGUGUGU; U6, Forward: CTCGCTTCGGCAGCACAU and Reverse: AACGCTTCACGAATTTGCGT. IGF1, Forward: GGCACTCTGC TTGCTCACCTTT and Reverse: CACGAATTGAAGAGCGTCCACC; PI3K, Forward: CTCAGGGAAAGCTGGACCAC and Reverse: TGGTTCAGACG AGCTTCTGTG; mTOR, Forward: ACCCAAGCCTGGGACCTCTA and Reverse: GGCTGGTTGGGGTCATATGTT; β-Actin, Forward: TCGTACCACTGGCATTG TGAT and Reverse: CGAAGTCTAGGGCAACATAGCA. The relative expression level of miRNA in the intestine and mRNA in cultured cells were determined by the 2^−ΔΔ*C*^_T_ method.

### Computational analysis

MiRNA target prediction was performed on-line using TargetScan (http://www.targetscan.org/vert_71/) and BiBiServ2 – RNAhybrid algorithms.

### Western blot

Intestine tissues frozen at −80°C were thawed and fully grinded into homogenate in lysis buffer containing protease inhibitors and phosphatase inhibitors in glass grinder. After lysing on ice for 30 min, the samples were centrifuged at 12,000 r/min for 15 min and supernatants were collected whose protein concentrations were detected by BCA Kit. Thirty microgram proteins of each sample were separated by 10% sodium dodecyl sulfate polyacrylamide gel electrophoresis (SDS-PAGE) and transferred to PVDF membranes. The membranes were then blocked with 5% nonfat milk in TBST at room temperature for 1 h and followed by incubation in primary antibody IGF1 (dilution in 1:1000, Cell Signaling Technology, Inc., U.S.A.), PI3K (dilution in 1:1000, Cell Signaling Technology, Inc., U.S.A.), Akt (dilution in 1:1000, Cell Signaling Technology, Inc., U.S.A.), mTOR (dilution in 1:1000, Cell Signaling Technology, Inc., U.S.A.), or GADPH (dilution in 1:5000, Cell Signaling Technology, Inc., U.S.A.) at 4°C overnight. For secondary antibody incubation, the membranes were first washed with TBST three times, 10 min each time, and then incubated with goat anti-rabbit secondary antibody (1:2000) at room temperature for 1 h. Target bands were illuminated by ECL developer and captured by gel imaging system. Absorbance value was analyzed by ImageJ (NIH, U.S.A.).

### Statistical analysis

Data were analyzed by SPSS 13.0 (SPSS Inc., U.S.A.) and are presented as mean ± standard error of the mean (SEM). Differences between multiple groups were compared by one-way ANOVA analysis with Tukey post-test. *P* value less than 0.05 was considered significant.

## Results

### Hydrogen-rich saline pretreatment attenuated intestinal ischemia reperfusion injury

**Figure 1:**
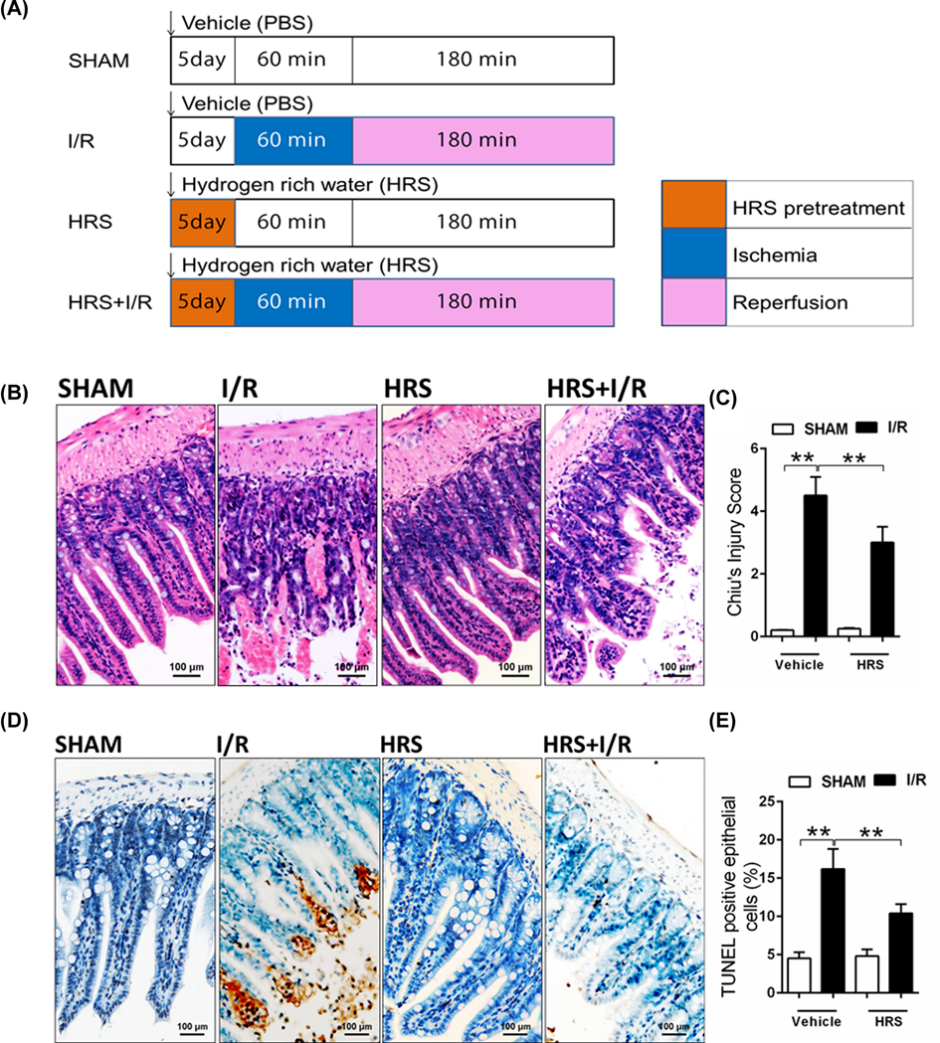
Experiment design and histology of intestinal I/R injury (**A**) Four experimental groups, SHAM, I/R, HRS, and HRS+I/R were designed in the present study. Mouse I/R model was established by 60 min of intestinal ischemia followed by 180 min of reperfusion. HRS (3 μmol/kg) was injected intraperitoneally once daily for five consecutive days before inducing intestinal I/R. The same volume of vehicle (saline) was given as control in SHAM and I/R groups. (**B**) Hematoxylin–eosin staining (×20) of intestine tissue and (**C**) grading of the observed injury based on Chiu’s score. (**D**) Intestinal epithelial cell apoptosis was detected by TUNEL assay (×40), and (**E**) TUNEL positive cells were quantified. Data are expressed as mean ± SEM, *n* = 8/group. **P < 0.01. HRS, hydrogen-rich saline; I/R, ischemia reperfusion; TUNEL, terminal deoxynucleotidyl transferase (TdT)-mediated dUTP nick end labeling.

To test the effects of HRS on intestinal I/R injury, daily i.p. injection of HRS (3 μmol/kg) was given for 5 days before inducing I/R by 60-min ischemia and 180-min reperfusion (Figure 1A). Histological change following I/R in the intestine visualized by hematoxylin–eosin staining demonstrated severe mucosal injury (Figure 1B). Compared with the normal mucosal villi in sham, I/R-injured intestine showed mucosal edema, small hemorrhagic spot on superficial mucosa, as well as necrotic and scaled superficial epithelium. HRS pretreatment did not disturb the normal mucosal structures in sham and preserved its integrity after I/R injury. Grading of mucosal damage by Chiu’s method also showed significantly lower score in the HRS-pretreated animals than the ones that had vehicle following I/R (*P* < 0.01, Figure 1C).

In addition, TUNEL assay revealed significantly increased apoptosis of mucosal epithelial cells in the I/R-injured intestine (*P* < 0.01 versus sham, Figure 1D,E). In contrast, HRS pretreatment markedly reduced the number of TUNEL positive epithelial cells (stained in brown) in the intestine after I/R (*P* < 0.01 versus I/R).

### Profiling of intestinal miRNAs in response to ischemia/reperfusion injury and pretreatment of hydrogen-rich saline

**Figure 2:**
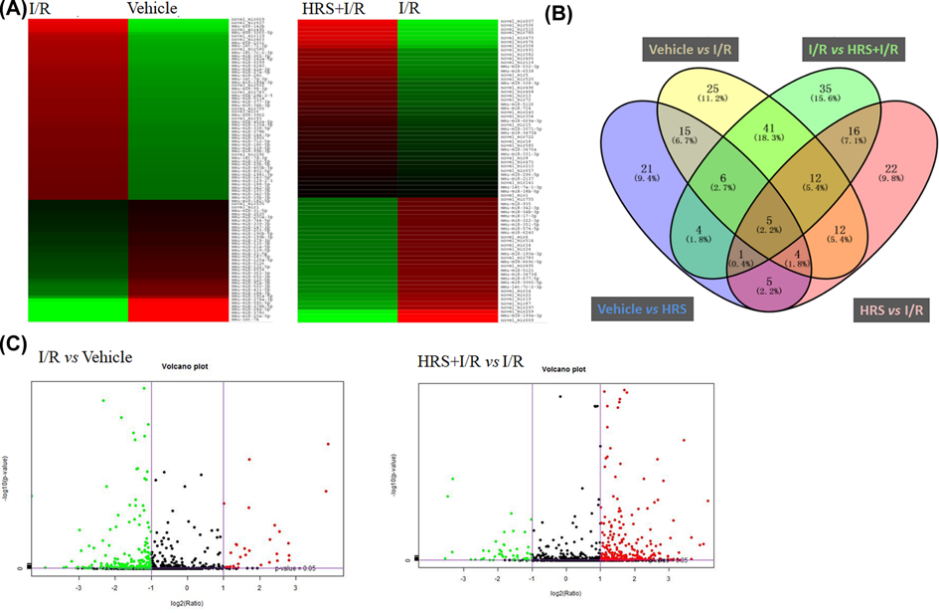
MiRNA sequencing analysis of intestinal mucosa Heat map (**A**) and volcano plots (**C**) present expression profiles of significantly altered miRNAs with fold change ≥2.0 (*P* < 0.05; false discovery rate <0.05) in response to I/R and HRS in the intestine. Green indicates down-regulation and red indicates up-regulation. Color brightness depicts the relative miRNAs expression level as brighter color represents higher expression. (**B**) Venn diagram indicates number of miRNAs counts in each group or shared between groups (overlapped area). HRS, hydrogen-rich saline; I/R, ischemia/reperfusion.

MiRNAs play a critical role in the pathophysiological process of intestinal I/R injury. To investigate novel targets for preventing mucosal injury after intestinal I/R, we performed miRNA sequencing of the I/R-injured intestine with or without HRS pretreatment. According to our cut-off criteria of *P* < 0.05 and fold-change ≥ 2.0, 120 differentially expressed miRNAs were revealed between I/R and sham groups, and between HRS+I/R and I/R groups, respectively (Figure 2A, data details see attachment Supplementary Tables S1 and S2). In comparison of I/R and sham groups, the number of down-regulated miRNAs was greater than the up-regulated ones, while the opposite case was seen in the comparison of HRS+I/R and I/R groups (Figure 2C). Of these 120 differentially expressed miRNAs found in each pair of comparison, 64 were shared between them (Figure 2B), which indicates the intestinal I/R injury-specific miRNAs altered by HRS pretreatment. We then identified the top 10 most diversely expressed miRNAs out of these 64 miRNAs and further selected 8 of them whose expression levels reached over 100 copies. These 8 miRNAs may be candidate targets for protection of intestine from IR injury: miR-199a-3p, miR-296-5p, miR-5126, miR- 6538, miR-342-3p, miR-677-5p, let-7c-2-3p, and miR-574-5p.

### RT-PCR validation of differentially expressed miRNAs

**Figure 3:**
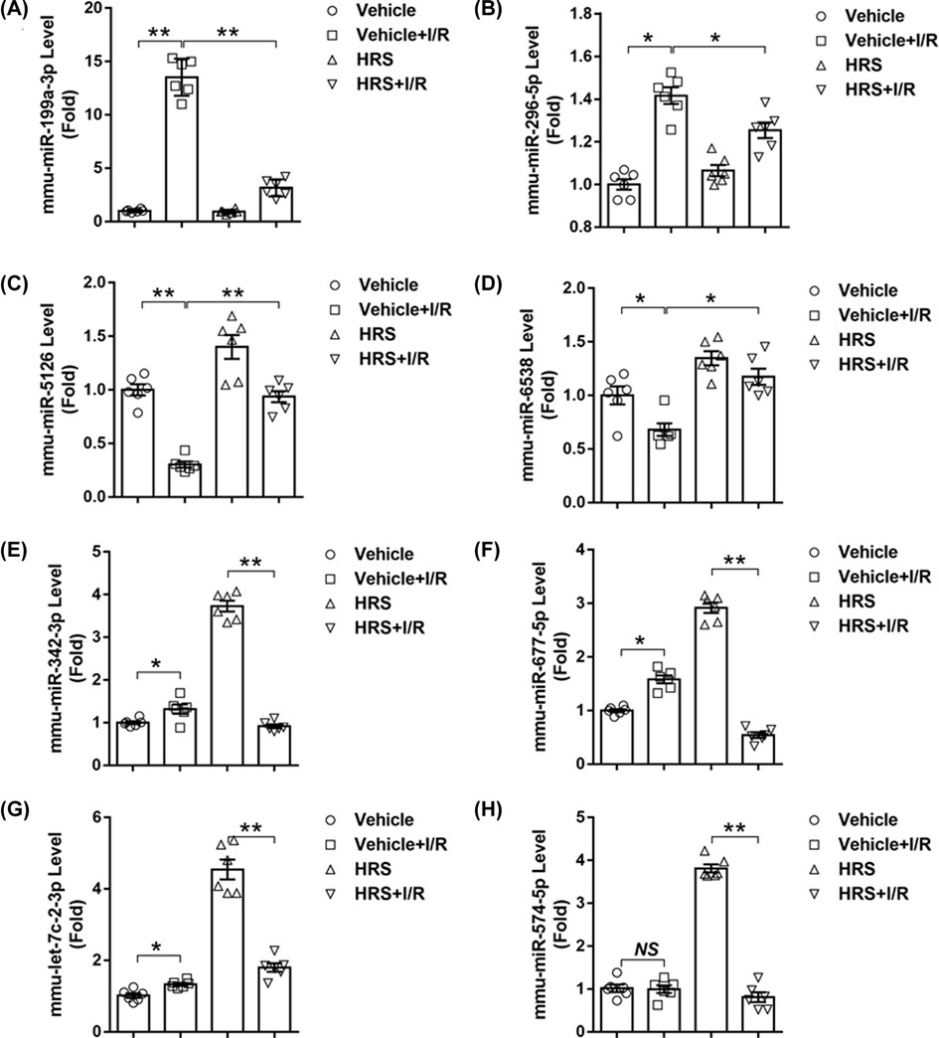
RT-PCR validation of candidate miRNAs responsive to HRS in intestinal I/R injury Expression levels of (**A**) mmu-miR-199a-3p, (**B**) mmu-miR-296-5p, (**C**) mmu-miR-5126, (**D**) mmu-miR-6538, (**E**) mmu-miR-342-3p, (**F**) mmu-miR-677-5p, (**G**) mmu-let-7c-2-3p, and (**H**) mmu-miR-574-5p are presented. Data are expressed as mean ± SEM, *n* = 6/group. *P < 0.05, **P < 0.01.

The eight chosen candidate miRNAs underwent validation of RT-PCR assay. Expressions of miR-199a-3p, miR-296-5p, miR-342-3p, miR-677-5p, and let-7c-2-3p were up-regulated (*P* < 0.05 versus sham, Figure 3A,B,E–G), while that of miR-5126 and miR- 6538 were down-regulated (*P* < 0.05 versus sham, Figure 3C,D) significantly after intestinal I/R. On the other hand, HRS pretreatment markedly down-regulated miR-199a-3p and miR-296-5p (*P* < 0.05 versus I/R, Figure 3A,B), but up-regulated miR-5126 and miR-6538 (*P* < 0.05 versus I/R, Figure 3C,D) following intestinal I/R; especially miR-5126 and miR- 6538 that were also up-regulated by HRS in sham animals. It’s worth noting that these four differentially expressed miRNAs in response to I/R with HRS pretreatment was oppositely regulated during I/R injury, indicating HRS could reverse these I/R-induced alterations. Expressions of miR-342-3p, miR-677-5p, mmu-let-7c-2-3p, and miR-574- 5p were not significantly altered between I/R and HRS+I/R groups (Figure 3E–H), denoting inconsistency with the miRNA microarray results.

### Predicted targets of miR-199a-3p is related to the IGF-1/PI3K/Akt/mTOR pathway

**Figure 4:**
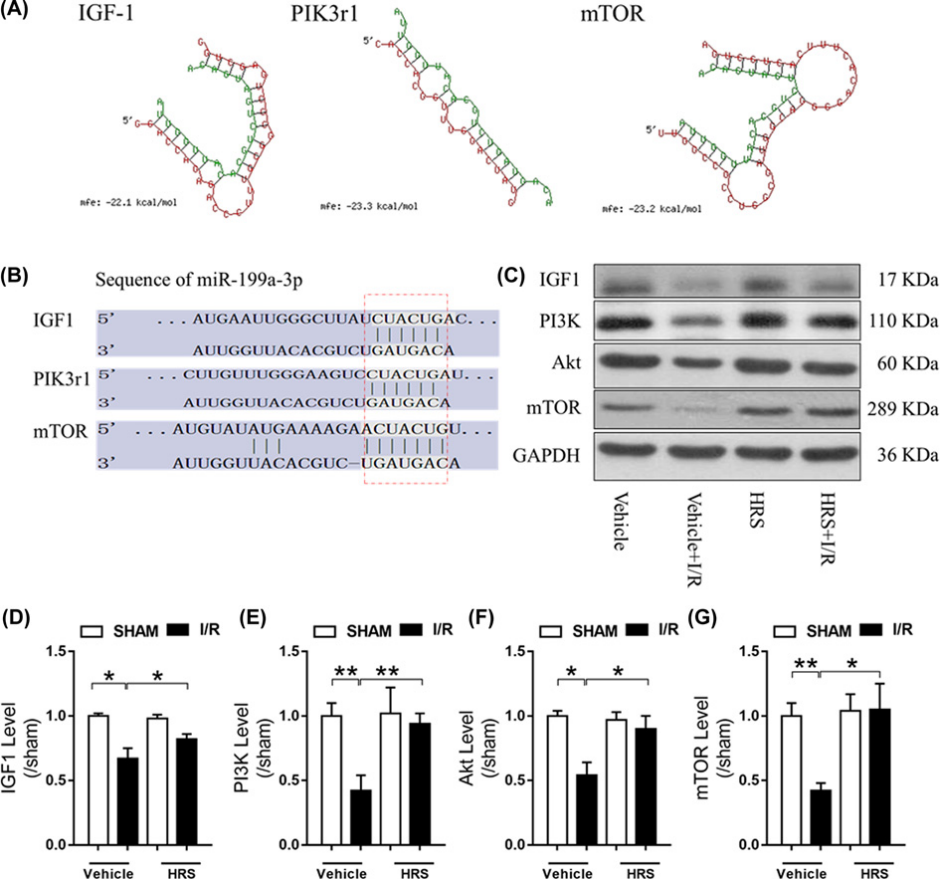
Predictive targets of mmu-miR-199a-3p and their related pathway (**A**) Schematic of miR-199a-3p binding to its target genes predicted by RNAhybrid. Green sequence represents miR-199a-3p mature sequence and the red represents its target gene. (**B**) Schematic of miR-199a-3p binding to its target genes predicted by TargetScan. (**C**) Representative images of IGF-1/PI3K/Akt/mTOR pathway proteins expressions and their quantification shown in (**D**) IGF-1, (**E**) PI3K, (**F**) Akt, and (**G**) mTOR. Data are expressed as mean ± SEM, *n* = 6/group. *P < 0.05, **P < 0.01. HRS, hydrogen-rich saline; I/R, ischemia/reperfusion; IGF-1, insulin-like growth factor-1; mTOR, mammalian target of rapamycin; PIK3r1, phosphoinositide-3-kinase regulatory subunit 1; PI3K, phosphoinositide-3-kinase.

Since miR-199a-3p displayed the most drastic change in response to I/R and HRS pretreatment among our validated miRNAs, we performed computational analysis of its potential targets and related pathways. Using RNAhybrid (Figure 4A) and TargetScan (Figure 4B) programs, we found that miR-199a-3p could target insulin-like growth factor (IGF)-1, mammalian target of rapamycin (mTOR), and phosphoinositide-3-kinase regulatory subunit 1 (PIK3r1), which is a gene coded for a key part of the phosphatidylinositol 3-kinase (PI3K). Interestingly, these three targets of miR-199a-3p form a well-known pathway that promotes cell growth and division, namely the IGF-1/PI3K/Akt/mTOR pathway [21,22]. Therefore, we examined the protein expression of this targeted pathway and found a general decrease in all four of its members’ expressions after intestinal I/R injury (*P* < 0.05 versus sham, Figure 4C–G). However, HRS pretreatment reduced these changes, significantly increasing protein levels of IGF-1, PI3K, Akt, and mTOR (*P* < 0.05 versus I/R).

### HRS protected intestinal epithelial cell from hypoxia/reoxygenation injury through down-regulating miR-199a-3p expression and reactivating mTOR-related pro-survival pathway

**Figure 5:**
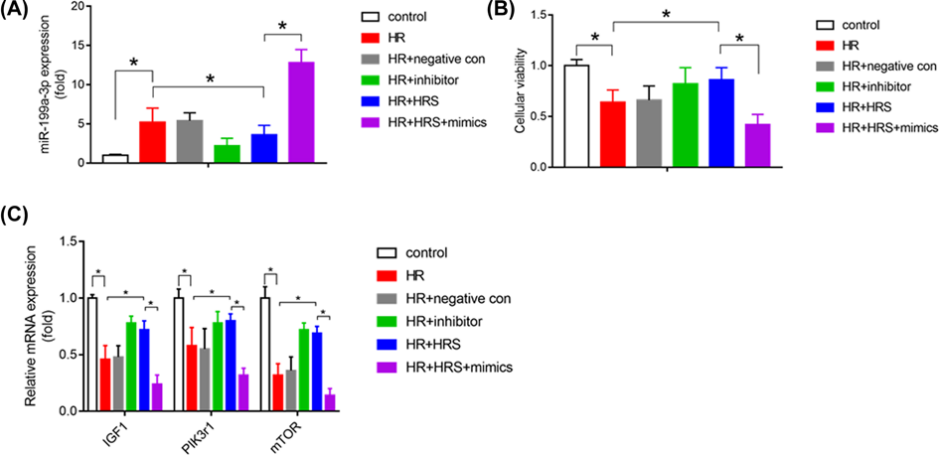
HRS protected intestinal epithelial cell from HR injury through down-regulating miR-199a-3p expression and reactivating mTOR-related pro-survival pathway (**A**) Relative miR-199a-3p expression was measured after HR with or without HRS treatment or transfecting with miR-199a-3p mimics or inhibitor in intestinal epithelial cell IEC-6. Cellular viability of IEC-6 was detected using CCK-8 method (**B**), and mRNA expression of IGF1, PIK3r1 and mTOR (**C**) were detected after HR with or without HRS or transfecting with miR-199a-3p mimics or inhibitor in IEC-6; n = 6. *P < 0.05. HR: hypoxia for 2 h/reoxygenation for 60 min; HR+negative con: miR- 199a-3p negative control was transfected to IEC-6 24 h before HR performed; HR+inhibitor: miR-199a-3p inhibitor was transfected to IEC-6 24 h before HR performed; HR+HRS: IEC-6 cells were pretreatment with HRS medium for 24 h before cells subjected to hypoxia/reoxygenation; HR+HRS+mimics: miR-199a-3p mimics was transfected to IEC-6 24 h before HRS treatment and subsequent HR performed.

In order to confirm the role of miR-199a-3p in intestinal I/R injury, we created intestinal epithelial cell IEC-6 hypoxia/reoxygenation to mimic *in vivo* intestinal I/R model. miR-199a-3p mimics or inhibitor were transfected into IEC-6 cells to perform the gain and loss functional experiments. As shown in Figure 5A–C, we found IEC-6 cells viability was significantly decreased after HR injury, accompany with decrease of IGF-1, PIK3r1, and mTOR mRNA expression. HRS treatment was found to reduce miR-199a-3p level, increase IGF-1, PIK3r1 and mTOR mRNA expression, restore IEC-6 cells viability, and this protective effects were similar to miR-199a-3p inhibitor treatment. Furthermore, the protective effects of HRS were reversed under miR-199a-3p mimics treatment. Taken together, aforementioned results suggest HRS treatment may protect intestinal epithelial cell from HR injury through down-regulating miR-199a-3p expression and activating IGF-1/PIK3r1/mTOR-related cell survival pathway.

## Discussion

**Figure 6:**
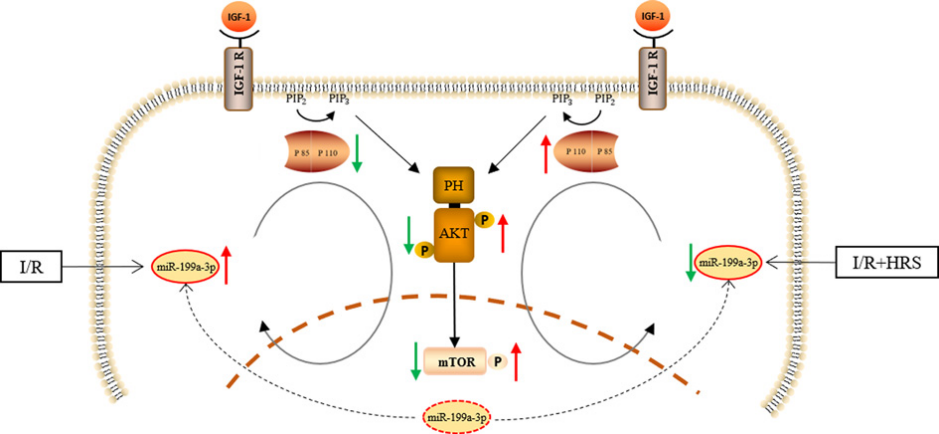
Schematic representation about the effect of HRS in intestinal I/R injury IGF-1/PI3K/Akt/mTOR pathway was downregulated during intestine I/R injury with miR-199a-3p significantly increase. HRS activated the IGF-1/PI3K/Akt/mTOR pathway via stimulating miR-199a-3p generation and attenuated the intestine I/R injury.

Our study identified and validated four novel miRNAs, miR-199a-3p, miR-296-5p, miR-5126, and miR-6538, which may mediate the protective effects of HRS in intestinal I/R injury. Further, we found that miR-199a-3p, the most significantly altered miRNA among these four, has its putative target gene linked to a cell growth-promoting pathway IGF-1/PI3K/Akt/mTOR [21], predicted by both RNAhybrid and TargetScan algorithms. The fact that miR-199a-3p was markedly increased by intestinal I/R is consistent with the significant decrease of IGF-1/PI3K/Akt/mTOR pathway protein expression following I/R, indicating potential suppression of the pro-survival pathway by miR-199a-3p. This is in line with the marked epithelial cell death and deteriorated mucosa in the intestine after I/R. HRS pretreatment effectively reduced miR-199a-3p and reversed these changes, suggesting a putative anti-apoptotic mechanism induced by HRS (Figure 6).

From hydrogen gas to HRS, hydrogen as a potent and selective antioxidant has been applied in clinic and studied in various animal disease models, especially in the setting of I/R injury [23–25]. As a well-established key feature of I/R injury, oxidative stress mediated by highly toxic ROS like hydroxyl radicals and peroxynitrite has been a main therapeutic target in interventions for I/R injury. For oxidative stress not only exaggerates host inflammatory response to I/R, but also damages cellular components indiscriminately, leading to escalated cellular apoptosis and thus tissue deterioration [5]. In line with previous studies [3,9], we demonstrated advanced disruption of intestinal mucosa in mice subjected to I/R, evidenced by massive apoptosis of epithelial cells and lifting of epithelium down the villi. However, mice pretreated with HRS exhibited significantly lower level of epithelial apoptosis and preserved tissue integrity in the intestine mucosa. These benefits have been documented previously and are attributable to the selective scavenging ability of HRS for hydroxyl radicals and peroxynitrite, effectively reducing oxidative stress and associated cell death [5,6]. Nonetheless, the prominent anti-apoptotic property of HRS may be mediated by more than just antioxidation.

Recent highlights of miRNAs in intestinal I/R injury pointed out a close link between miRNAs and I/R-induced oxidative stress, inflammation, and cell death [10,11,14]. Indeed, by negatively regulate gene expressions, miRNAs stay in controls of highly regulated events in cell, such as cellular proliferation, differentiation, and apoptosis [12]. Therefore, we profiled miRNAs that were expressed differentially in intestinal I/R and validated the ones that were most responsive to HRS pretreatment. We found that intestinal I/R significantly increased miR-199a-3p and miR-296-5p levels, and decreased miR-5126 and miR-6538 levels, which can be prevented by HRS. Of these four, miR-296-5p, miR-5126, and miR-6538 has been reported in intestinal-related illness. They were found significantly altered in the intestinal epithelial cells during inflammatory bowel diseases [26]. It is also known that miR-296-5p regulates genes involved in inflammation, angiogenesis, hypertension, cholesterol metabolism, cellular proliferation, and apoptosis. Previous literature suggested it to be a promising target of atherosclerosis, an inflammatory disease in the vascular wall [27]. Given the disturbances in blood circulation and the inflammatory nature of I/R injury, it is reasonable to defer that miR-296-5p may be a key contributor to the pathogenesis of intestinal I/R injury.

On the other hand, miR-5126 and miR-6538 has been demonstrated responsive to blood-stage malaria infection, while miR-5126 was confirmed down-regulated in macrophage infected with bacteria *Brucella* [28]. Of note, they were both down-regulated in exosome extracted from plasma after I/R preconditioning in the abdominal aorta [29], which resembles a mild version of I/R injury in the abdominal circulation. These findings support our result that miR-5126 and miR-6538 decreased in response to intestinal I/R injury and may also be associated with the I/R-induced bacterial translocation in the circulation. Therefore, HRS may prevent intestinal I/R injury by prophylactically up-regulate miR-5126 and miR-6538, as seen in HRS-pretreated sham animals, which then afforded them the later decrease by I/R, hence reduced injury.

Regarding miR-199a-3p, the most drastically altered miRNA in the miRNomes in HRS-pretreated intestinal I/R injury, has never been reported in intestinal diseases. Interestingly, miR-199a-3p, miR-5126, and miR-6538 have been listed together among the top differentially expressed miRNAs in mitochondria from the failing heart at late stage, suggesting link to energy metabolism, oxidative stress, and apoptosis [30]. Specifically, study has highlighted that miR-199a-3p inhibits cell proliferation and induces apoptosis in human hepatocellular carcinoma [31]. These are in line with our current finding. Target prediction of miR-199a-3p revealed involvement of the cell survival pathway, IGF-1/PI3K/Akt/mTOR [21,22], suggesting miR-199a-3p may suppress the pathway and induce apoptosis. Further pathway protein analysis showed decreases in IGF-1/PI3K/Akt/mTOR levels after intestinal I/R, which agreed with the up-regulation of miR-199a-3p following the injury, supporting the notion on the inhibition of IGF-1/PI3K/Akt/mTOR by miR-199a-3p. In *in vitro* experiment, IEC-6 HR model was created to mimic *in vivo* intestinal I/R, HRS or miR-199a-3p inhibitor treatment were found to decrease miR-199a-3p level, increase IGF-1, PIK3r1, and mTOR mRNA expression. However, the effects of HRS were reversed under miR- 199a-3p mimics treatment. The fact that these changes together with epithelial cell apoptosis in the I/R-injured intestine can be reversed by HRS-pretreatment indicated the anti-apoptotic effect of HRS may be mediated by regulating miR-199a-3p.

## Conclusion

In conclusion, our results revealed novel miRNAs involved in the protective mechanism of HRS in intestinal I/R injury. In particular, we demonstrated that miR- 199a-3p may serve a key role in the anti-apoptotic mechanism of HRS that contributes to its protection of the intestine against I/R injury.

## Supplementary Material

Supplementary Tables S1-S2

## Abbreviations

H&E: hematoxylin–eosin
HRS: hydrogen-rich saline
IGF: insulin-like growth factor
I/R: ischemia/reperfusion
miRNA: microRNA
mTOR: mammalian target of rapamycin
PI3K: phosphoinositide-3-kinase
PIK3r1: phosphoinositide-3-kinase regulatory subunit 1
ROS: reactive oxygen species
SMA: superior mesenteric artery
TUNEL: (TdT)-mediated dUTP nick end labeling
