# Supplementary material for: MicroRNA files in the prevention of intestinal ischemia/reperfusion injury by hydrogen rich saline
Source: Biosci Rep. 2020 Jan 24;40(1):BSR20191043. doi: 10.1042/BSR20191043 (PMC6981100; doi:10.1042/BSR20191043)
Supplement: Supplementary Tables S1-S2 [file BSR-2019-1043_supp.pdf]

Supple Table 1: Differential miRNAs between Sham and IR groups

miRNA id	Read count(Sham)	Read count(IR)	Expression(Sham)	Expression(IR)	log2Ratio(IR/Sham)	Up down regulation
mmu-miR-21a-5p	757386	519731	9599.666667	5186.77	-1.431266883	DOWN
mmu-miR-378a-3p	87506	7355	1138.303333	80.32333333	-4.460587937	DOWN
mmu-miR-200a-3p	141382	122238	1757.086667	1217.28	-1.097908854	DOWN
mmu-miR-378b	46622	190913	498.2366667	1909.353333	1.145829303	UP
mmu-miR-92a-3p	29394	11326	376.4833333	113.8133333	-2.263886504	DOWN
mmu-miR-652-3p	34257	18111	437.1366667	181.91	-1.80753585	DOWN
novel_mir1	40097	32918	466.01	349.0333333	-1.172620858	DOWN
mmu-miR-199b-3p	29718	20907	374.47	221.6166667	-1.395354208	DOWN
novel_mir93	14922	64824	168.2633333	699.6566667	1.23108389	UP
mmu-miR-3074-5p	6140	57	79.08666667	0.58	-7.639136168	DOWN
mmu-miR-5126	12467	6918	151.46	71.11	-1.737690667	DOWN
mmu-miR-150-5p	10112	41722	126.07	433.9633333	1.156736706	UP
mmu-miR-142a-5p	2679	18187	31.57666667	187.3133333	1.875137858	UP
mmu-miR-142b	877	11095	9.563333333	114.01	2.773185773	UP
mmu-miR-31-5p	22781	20572	278.1833333	203.6466667	-1.03515226	DOWN
mmu-miR-199a-3p	3984	21854	43.25666667	210.5633333	1.56760457	UP
novel_mir755	4959	24062	53.71666667	233.72	1.39063219	UP
novel_mir807	4123	661	54.72	6.743333333	-3.528975526	DOWN
mmu-miR-33-5p	8622	5413	108.0266667	54.32333333	-1.559597416	DOWN
mmu-miR-28c	2840	15292	30.24033333	154.94	1.540811028	UP
mmu-miR-28a-5p	2448	82	31.82333333	0.833333333	-5.78783908	DOWN
mmu-miR-125a-5p	6866	4086	86.67666667	41.34666667	-1.636783895	DOWN
mmu-let-7k	2189	43	28.03333333	0.426666667	-6.557794726	DOWN
mmu-miR-378d	2141	10	28.557	0.110666667	-8.630144227	DOWN
mmu-miR-802-5p	4532	17434	51.86	175.21	1.055680606	UP
mmu-miR-20a-5p	1620	42	20.71	0.423333333	-6.157463917	DOWN
novel_mir669	273	4141	2.936666667	39.70666667	3.035003104	UP
mmu-let-7d-3p	3744	14926	43.47666667	149.9133333	1.107172009	UP
mmu-miR-182-5p	8246	7246	96.61666667	73.39	-1.074512897	DOWN
mmu-miR-5099	1103	6953	12.16666667	70.71	1.768199557	UP
mmu-miR-139-5p	4757	3159	59.55333333	31.86666667	-1.47858733	DOWN
mmu-miR-374c-5p	4	2938	0.050333333	28.85666667	8.632615439	UP
mmu-miR-196a-5p	1761	408	22.77666667	4.166666667	-2.997757094	DOWN
mmu-miR-6538	3255	1784	39.63666667	17.96666667	-1.75554517	DOWN
novel_mir296	3432	12944	36.73666667	127.98	1.027158813	UP
mmu-let-7c-2-3p	746	4722	7.953333333	48.01333333	1.774147264	UP

mmu-miR-342-3p	2368	9281	28.72	93.40666667	1.082607937	UP
mmu-miR-20b-5p	2076	8446	24.72	84.74333333	1.136458561	UP
mmu-miR-374b-5p	701	33	9.136666667	0.336666667	-5.296879757	DOWN
novel_mir495	160	1872	1.82	19.73	2.660433383	UP
mmu-let-7i-3p	288	2174	3.496666667	22.20333333	2.028207982	UP
novel_mir14	23	900	0.244	9.536666667	4.402215993	UP
novel_mir502	505	2655	5.736666667	27.74333333	1.506353326	UP
novel_mir13	478	53	5.087	0.507333333	-4.060949595	DOWN
novel_mir119	204	1627	2.373333333	16.89666667	2.107569952	UP
mmu-miR-378c	409	13	5.320333333	0.13	-5.863520557	DOWN
novel_mir785	378	44	4.876666667	0.443666667	-3.990814048	DOWN
mmu-miR-101c	208	1426	2.263333333	14.46	1.889315306	UP
novel_mir506	39	740	0.463666667	8.14	3.357976	UP
mmu-miR-365-3p	201	1373	2.38	14.20333333	1.884060977	UP
mmu-miR-21a-3p	233	1409	2.773333333	14.37	1.70826651	UP
novel_mir6	383	1867	4.4	18.94666667	1.397302388	UP
novel_mir151	481	189	5.556666667	1.826666667	-2.235653902	DOWN
mmu-miR-186-5p	465	2016	5.413333333	20.35333333	1.228189776	UP
mmu-let-7j	256	2	3.297	0.020666667	-7.888003242	DOWN
mmu-miR-187-3p	605	346	7.513333333	3.696666667	-1.694166346	DOWN
novel_mir297	71	696	0.757333333	6.663666667	2.405193134	UP
novel_mir8	718	494	8.23	5.066666667	-1.427476044	DOWN
mmu-miR-135a-5p	362	1583	4.173333333	16.19	1.240596411	UP
mmu-miR-15b-3p	599	2219	7.05	22.41666667	1.001278517	UP
novel_mir259	135	826	1.437333333	7.917	1.725179132	UP
novel_mir12	450	262	4.91	2.540333333	-1.668361432	DOWN
mmu-miR-122-5p	330	146	4.296666667	1.473333333	-2.064500897	DOWN
mmu-miR-500-3p	274	99	3.51	1.003333333	-2.356678705	DOWN
mmu-miR-363-3p	385	205	4.806666667	2.08	-1.797237778	DOWN
novel_mir496	14	289	0.150666667	3.12	3.479567518	UP
mmu-miR-592-5p	126	7	1.633333333	0.073333333	-5.057928243	DOWN
novel_mir743	167	822	1.813666667	8.366666667	1.411287049	UP
novel_mir607	28	344	0.297333333	3.743333333	2.730906591	UP
mmu-miR-3065-5p	48	423	0.513666667	4.243333333	2.25154811	UP
mmu-miR-467e-5p	123	1	1.560333333	0.010666667	-7.830517747	DOWN
novel_mir228	0	228	0.001	2.263666667	7.944886772	UP
mmu-miR-3962	188	850	2.01	8.596666667	1.288726937	UP
mmu-miR-132-3p	425	311	5.216666667	3.08	-1.338551503	DOWN

mmu-miR-6240	80	501	0.91	4.993333333	1.758735456	UP
novel_mir418	148	39	1.577333333	0.377	-2.812054389	DOWN
novel_mir656	0	183	0.001	1.744	7.627696596	UP
mmu-miR-450a-5p	136	646	1.586666667	6.543333333	1.359924271	UP
novel_mir657	14	220	0.150666667	2.416666667	3.08600155	UP
novel_mir5	81	467	0.886666667	4.93	1.639425495	UP
mmu-miR-99b-3p	212	844	2.416666667	8.576666667	1.105175492	UP
mmu-miR-184-3p	135	41	1.446666667	0.406666667	-2.607266834	DOWN
novel_mir463	56	375	0.597333333	4.040333333	1.855388621	UP
novel_mir514	12	200	0.127333333	1.910333333	3.170890447	UP
mmu-miR-431-5p	143	51	1.853333333	0.526666667	-2.375449237	DOWN
mmu-miR-27a-5p	65	392	0.766666667	3.93	1.704338789	UP
mmu-miR-744-5p	336	264	3.9	2.68	-1.235926545	DOWN
novel_mir585	50	338	0.563666667	3.463333333	1.869020005	UP
mmu-miR-712-5p	168	686	1.9	7.193333333	1.141744101	UP
novel_mir141	331	262	3.996666667	2.676666667	-1.225267647	DOWN
mmu-miR-3535	315	243	3.476666667	2.416666667	-1.262398757	DOWN
mmu-miR-132-5p	205	122	2.606666667	1.213333333	-1.636746004	DOWN
novel_mir392	74	0	0.817	0.001	-8.097456608	DOWN
novel_mir508	223	149	2.666666667	1.546666667	-1.469734621	DOWN
novel_mir722	0	111	0.001	1.16	6.906412624	UP
mmu-miR-376b-5p	131	61	1.683333333	0.616666667	-1.990688906	DOWN
mmu-miR-144-3p	104	464	1.253333333	4.78	1.269538035	UP
novel_mir22	99	440	1.054	4.197333333	1.263999851	UP
novel_mir473	90	28	1.150333333	0.300333333	-2.572501416	DOWN
novel_mir51	0	103	0.001	0.993666667	6.798497285	UP
novel_mir452	0	100	0.001	0.954	6.755852948	UP
novel_mir663	42	261	0.447	2.523333333	1.747585332	UP
novel_mir49	95	34	1.010666667	0.324	-2.370396009	DOWN
mmu-miR-196b-5p	169	109	1.873333333	1.12	-1.520698353	DOWN
novel_mir619	0	96	0.001	0.930333333	6.696959259	UP
mmu-miR-380-3p	72	17	0.91	0.17	-2.970465402	DOWN
novel_mir600	0	94	0.001	0.897333333	6.66658561	UP
mmu-miR-107-3p	74	20	0.946666667	0.196666667	-2.775528513	DOWN
mmu-miR-98-3p	62	314	0.7	3.23	1.452421197	UP
novel_mir640	0	89	0.001	0.896666667	6.587730189	UP
novel_mir87	79	362	0.947	4.010333333	1.308061897	UP
mmu-miR-29a-5p	80	27	1.023333333	0.273333333	-2.455043835	DOWN

novel_mir729	0	85	0.001	0.810666667	6.521387694	UP
novel_mir350	48	0	0.510666667	0.001	-7.472965743	DOWN
mmu-miR-29b-2-5	52	274	0.596666667	2.743333333	1.509589123	UP
novel_mir48	70	327	0.747333333	3.140333333	1.335860567	UP
mmu-miR-411-3p	50	6	0.64	0.063666667	-3.946896931	DOWN
novel_mir766	0	82	0.001	0.784	6.469548763	UP
novel_mir38	0	81	0.001	0.774	6.451846761	UP
novel_mir599	0	80	0.001	0.777	6.433924853	UP
novel_mir460	44	0	0.587333333	0.001	-7.347434861	DOWN
novel_mir237	0	76	0.001	0.753333333	6.359924271	UP
novel_mir802	43	0	0.496666667	0.001	-7.314267997	DOWN
novel_mir39	11	121	0.117333333	1.154	2.571428377	UP
novel_mir162	41	0	0.47	0.001	-7.245555247	DOWN
novel_mir231	95	49	1.047	0.477	-1.843149006	DOWN
mmu-miR-92a-1-5	18	145	0.216666667	1.42	2.121980847	UP
novel_mir771	137	96	1.49	0.923666667	-1.401072824	DOWN
novel_mir31	28	178	0.297333333	1.753333333	1.780375267	UP
novel_mir34	20	150	0.214	1.430666667	2.018887354	UP
mmu-miR-7662-3p	36	2	0.473666667	0.020333333	-5.057928243	DOWN
novel_mir573	37	0	0.394	0.001	-7.097456608	DOWN
novel_mir751	0	65	0.001	0.620666667	6.134364571	UP
novel_mir649	36	0	0.384	0.001	-7.057928243	DOWN
mmu-miR-669o-5p	34	1	0.437	0.010666667	-5.975466083	DOWN
novel_mir511	0	63	0.001	0.600666667	6.089276682	UP
mmu-miR-339-5p	77	317	0.903333333	3.266666667	1.153549247	UP
novel_mir481	35	0	0.374	0.001	-7.017286259	DOWN
novel_mir689	37	199	0.394	1.897333333	1.539168013	UP
novel_mir96	0	61	0.001	0.580666667	6.042734096	UP
mmu-miR-338-3p	85	45	1.02	0.493333333	-1.805541082	DOWN
novel_mir795	53	16	0.597	0.154	-2.615923697	DOWN
novel_mir571	0	60	0.001	0.574	6.018887354	UP
mmu-miR-147-3p	160	132	1.98	1.313333333	-1.165537217	DOWN
novel_mir482	0	59	0.001	0.600666667	5.994639807	UP
mmu-miR-690	50	15	0.566666667	0.153333333	-2.624968836	DOWN
novel_mir444	0	57	0.001	0.544	5.944886772	UP
mmu-miR-541-5p	92	56	1.126666667	0.566666667	-1.604210276	DOWN
novel_mir109	61	261	0.650666667	2.753666667	1.209165417	UP
novel_mir445	31	0	0.330666667	0.001	-6.842199552	DOWN

novel_mir465	0	55	0.001	0.597	5.893356472	UP
novel_mir30	29	0	0.310666667	0.001	-6.745984237	DOWN
mmu-miR-338-5p	52	230	0.603333333	2.33	1.257047091	UP
novel_mir247	28	0	0.297333333	0.001	-6.695358164	DOWN
novel_mir621	21	133	0.224	1.277	1.774961771	UP
novel_mir190	0	50	0.001	0.477333333	5.755852948	UP
mmu-miR-143-5p	59	246	0.746666667	2.496666667	1.171868214	UP
mmu-miR-203-5p	117	90	1.426666667	0.906666667	-1.266514865	DOWN
novel_mir405	82	50	0.874	0.490333333	-1.601699057	DOWN
mmu-miR-23b-5p	37	182	0.433333333	1.803333333	1.410338033	UP
novel_mir545	88	57	0.973666667	0.557	-1.514544846	DOWN
novel_mir275	27	0	0.287333333	0.001	-6.642890744	DOWN
novel_mir114	0	48	0.001	0.457333333	5.696959259	UP
novel_mir386	20	126	0.214	1.200666667	1.767348587	UP
novel_mir746	26	0	0.277	0.001	-6.58844296	DOWN
mmu-miR-342-5p	74	281	0.89	2.846666667	1.036969713	UP
mmu-miR-1843a-3	51	21	0.636666667	0.216666667	-2.168111161	DOWN
novel_mir333	25	0	0.334	0.001	-6.531859432	DOWN
novel_mir410	88	60	0.937333333	0.574	-1.440544265	DOWN
mmu-miR-330-5p	99	73	1.24	0.726666667	-1.327535303	DOWN
novel_mir559	17	113	0.180666667	1.087	1.844712879	UP
novel_mir576	0	45	0.001	0.480333333	5.603849854	UP
novel_mir54	49	20	0.557	0.207	-2.180784991	DOWN
mmu-miR-5113	37	175	0.436666667	1.75	1.353754504	UP
novel_mir154	0	44	0.001	0.420666667	5.571428377	UP
novel_mir158	24	0	0.290333333	0.001	-6.472965743	DOWN
novel_mir107	24	0	0.257333333	0.001	-6.472965743	DOWN
novel_mir712	0	43	0.001	0.413666667	5.538261513	UP
novel_mir441	0	43	0.001	0.410666667	5.538261513	UP
novel_mir258	0	43	0.001	0.410666667	5.538261513	UP
novel_mir149	0	42	0.001	0.400666667	5.504314181	UP
novel_mir491	0	41	0.001	0.457333333	5.469548763	UP
novel_mir588	22	0	0.234	0.001	-6.347434861	DOWN
mmu-miR-466f	10	82	0.106666667	0.836666667	2.147620668	UP
mmu-miR-188-5p	57	223	0.686666667	2.243333333	1.080006644	UP
novel_mir680	71	46	0.810333333	0.483666667	-1.514188405	DOWN
mmu-miR-100-3p	66	246	0.783333333	2.52	1.010117144	UP
novel_mir312	10	81	0.107333333	0.843333333	2.129918666	UP

novel_mir246	92	71	1.013666667	0.707	-1.261818079	DOWN
mmu-miR-183-3p	35	11	0.453333333	0.107	-2.55785464	DOWN
novel_mir811	21	0	0.224	0.001	-6.280320665	DOWN
novel_mir759	21	0	0.224	0.001	-6.280320665	DOWN
novel_mir284	21	0	0.224	0.001	-6.280320665	DOWN
mmu-miR-329-3p	62	234	0.753333333	2.356666667	1.028165167	UP
novel_mir505	125	113	1.447	1.090333333	-1.033608564	DOWN
novel_mir770	0	38	0.001	0.364	5.359924271	UP
novel_mir321	0	38	0.001	0.364	5.359924271	UP
novel_mir534	0	38	0.001	0.364	5.359924271	UP
novel_mir41	33	10	0.350666667	0.097333333	-2.610469266	DOWN
mmu-miR-450b-3p	56	216	0.64	2.166666667	1.059529338	UP
novel_mir800	20	0	0.214	0.001	-6.209931337	DOWN
novel_mir736	20	0	0.214	0.001	-6.209931337	DOWN
novel_mir772	20	0	0.214	0.001	-6.209931337	DOWN
novel_mir428	0	37	0.001	0.414	5.321450124	UP
mmu-miR-335-3p	30	142	0.346666667	1.466666667	1.354853282	UP
novel_mir697	19	0	0.204	0.001	-6.135930755	DOWN
mmu-miR-467h	19	0	0.254	0.001	-6.135930755	DOWN
novel_mir609	19	0	0.204	0.001	-6.135930755	DOWN
mmu-miR-377-3p	25	125	0.286666667	1.263333333	1.433924853	UP
novel_mir252	0	35	0.001	0.360333333	5.241279775	UP
novel_mir101	0	35	0.001	0.334	5.241279775	UP
novel_mir652	0	35	0.001	0.334	5.241279775	UP
novel_mir557	0	35	0.001	0.356666667	5.241279775	UP
novel_mir403	0	35	0.001	0.334	5.241279775	UP
mmu-miR-1981-3p	54	206	0.613666667	2.056666667	1.043609783	UP
novel_mir388	31	10	0.330666667	0.097333333	-2.520271457	DOWN
novel_mir400	0	34	0.001	0.324	5.199459599	UP
novel_mir148	0	34	0.001	0.324	5.199459599	UP
mmu-miR-339-3p	85	68	1.03	0.693333333	-1.209931337	DOWN
novel_mir574	82	65	0.943666667	0.647	-1.223187434	DOWN
novel_mir40	15	91	0.160666667	0.880333333	1.712900803	UP
mmu-miR-331-3p	55	34	0.723333333	0.37	-1.581900114	DOWN
novel_mir198	0	33	0.001	0.314	5.156390877	UP
novel_mir60	0	33	0.001	0.314	5.156390877	UP
novel_mir61	0	33	0.001	0.314	5.156390877	UP
novel_mir346	0	33	0.001	0.314	5.156390877	UP

novel_mir562	0	33	0.001	0.314	5.156390877	UP
novel_mir52	51	31	0.544	0.297333333	-1.606232274	DOWN
mmu-miR-467b-5p	35	15	0.433333333	0.160333333	-2.110395663	DOWN
novel_mir666	0	31	0.001	0.314	5.066193068	UP
novel_mir435	16	0	0.170666667	0.001	-5.888003242	DOWN
novel_mir408	16	0	0.170666667	0.001	-5.888003242	DOWN
novel_mir731	0	30	0.001	0.287333333	5.018887354	UP
novel_mir715	0	30	0.001	0.287333333	5.018887354	UP
novel_mir782	0	30	0.001	0.287333333	5.018887354	UP
novel_mir99	0	30	0.001	0.287333333	5.018887354	UP
novel_mir568	0	30	0.001	0.287333333	5.018887354	UP
mmu-miR-379-3p	59	42	0.733333333	0.433333333	-1.378328869	DOWN
mmu-miR-34b-3p	19	98	0.226666667	0.993333333	1.478779089	UP
novel_mir421	0	29	0.001	0.277333333	4.969977753	UP
novel_mir95	0	29	0.001	0.297	4.969977753	UP
novel_mir629	0	29	0.001	0.277333333	4.969977753	UP
novel_mir230	0	29	0.001	0.277333333	4.969977753	UP
novel_mir188	0	29	0.001	0.277333333	4.969977753	UP
novel_mir477	0	29	0.001	0.324	4.969977753	UP
novel_mir425	0	29	0.001	0.277333333	4.969977753	UP
mmu-miR-129-2-3	41	160	0.496666667	1.623333333	1.076372848	UP
novel_mir612	15	0	0.160666667	0.001	-5.794893838	DOWN
novel_mir719	15	0	0.160666667	0.001	-5.794893838	DOWN
novel_mir301	15	0	0.160666667	0.001	-5.794893838	DOWN
novel_mir337	15	0	0.160666667	0.001	-5.794893838	DOWN
novel_mir78	0	28	0.001	0.277	4.91935168	UP
novel_mir165	0	28	0.001	0.267333333	4.91935168	UP
novel_mir398	0	28	0.001	0.314	4.91935168	UP
novel_mir671	0	28	0.001	0.267333333	4.91935168	UP
novel_mir610	0	28	0.001	0.267333333	4.91935168	UP
novel_mir335	0	27	0.001	0.257333333	4.86688426	UP
novel_mir493	0	27	0.001	0.263666667	4.86688426	UP
novel_mir490	0	27	0.001	0.257333333	4.86688426	UP
novel_mir222	0	27	0.001	0.257333333	4.86688426	UP
novel_mir123	0	27	0.001	0.257333333	4.86688426	UP
novel_mir613	0	27	0.001	0.257333333	4.86688426	UP
novel_mir563	0	27	0.001	0.257333333	4.86688426	UP
novel_mir376	0	27	0.001	0.257333333	4.86688426	UP

novel_mir218	14	79	0.187333333	0.860333333	1.608422584	UP
novel_mir659	38	21	0.404	0.200666667	-1.743613333	DOWN
novel_mir777	14	0	0.150666667	0.001	-5.695358164	DOWN
novel_mir298	14	0	0.150666667	0.001	-5.695358164	DOWN
novel_mir739	14	0	0.150666667	0.001	-5.695358164	DOWN
novel_mir803	14	0	0.150666667	0.001	-5.695358164	DOWN
novel_mir254	14	0	0.150666667	0.001	-5.695358164	DOWN
novel_mir792	14	0	0.150666667	0.001	-5.695358164	DOWN
novel_mir546	14	0	0.150666667	0.001	-5.695358164	DOWN
novel_mir768	55	40	0.587333333	0.380666667	-1.347434861	DOWN
novel_mir323	0	26	0.001	0.247333333	4.812436476	UP
novel_mir26	0	26	0.001	0.247333333	4.812436476	UP
novel_mir429	0	26	0.001	0.247333333	4.812436476	UP
novel_mir728	0	26	0.001	0.247333333	4.812436476	UP
novel_mir223	0	26	0.001	0.290666667	4.812436476	UP
novel_mir443	0	26	0.001	0.267	4.812436476	UP
novel_mir565	15	80	0.160666667	0.853666667	1.527034257	UP
novel_mir159	0	25	0.001	0.240666667	4.755852948	UP
novel_mir307	0	25	0.001	0.240666667	4.755852948	UP
novel_mir79	0	25	0.001	0.240666667	4.755852948	UP
novel_mir705	0	25	0.001	0.240666667	4.755852948	UP
novel_mir91	0	25	0.001	0.240666667	4.755852948	UP
novel_mir810	13	0	0.140666667	0.001	-5.58844296	DOWN
novel_mir205	13	0	0.140666667	0.001	-5.58844296	DOWN
novel_mir801	13	0	0.140666667	0.001	-5.58844296	DOWN
novel_mir168	26	11	0.277333333	0.124	-2.129011341	DOWN
novel_mir303	0	24	0.001	0.230666667	4.696959259	UP
novel_mir110	0	24	0.001	0.230666667	4.696959259	UP
novel_mir776	0	24	0.001	0.230666667	4.696959259	UP
mmu-miR-125b-1-	70	62	0.873333333	0.636666667	-1.063089949	DOWN
novel_mir488	12	0	0.127333333	0.001	-5.472965743	DOWN
novel_mir315	12	0	0.127333333	0.001	-5.472965743	DOWN
mmu-miR-3058-3p	12	0	0.147	0.001	-5.472965743	DOWN
novel_mir140	12	0	0.127333333	0.001	-5.472965743	DOWN
novel_mir194	12	0	0.127333333	0.001	-5.472965743	DOWN
novel_mir219	0	23	0.001	0.220666667	4.635558714	UP
novel_mir699	0	23	0.001	0.220666667	4.635558714	UP
novel_mir385	0	23	0.001	0.220666667	4.635558714	UP

novel_mir115	0	22	0.001	0.210666667	4.571428377	UP
novel_mir64	0	22	0.001	0.210666667	4.571428377	UP
novel_mir150	0	22	0.001	0.210666667	4.571428377	UP
novel_mir507	0	22	0.001	0.210666667	4.571428377	UP
novel_mir76	0	22	0.001	0.210666667	4.571428377	UP
novel_mir781	0	22	0.001	0.210666667	4.571428377	UP
novel_mir245	11	0	0.147333333	0.001	-5.347434861	DOWN
novel_mir305	11	0	0.117333333	0.001	-5.347434861	DOWN
novel_mir324	11	0	0.117333333	0.001	-5.347434861	DOWN
novel_mir328	11	0	0.117333333	0.001	-5.347434861	DOWN
novel_mir127	11	0	0.117333333	0.001	-5.347434861	DOWN
novel_mir238	11	0	0.117333333	0.001	-5.347434861	DOWN
novel_mir344	11	0	0.117333333	0.001	-5.347434861	DOWN
mmu-miR-154-3p	6	45	0.07	0.466666667	2.018887354	UP
mmu-miR-190a-3p	15	74	0.173666667	0.74	1.414559528	UP
novel_mir628	0	21	0.001	0.200666667	4.504314181	UP
novel_mir248	0	21	0.001	0.200666667	4.504314181	UP
novel_mir84	0	21	0.001	0.214	4.504314181	UP
novel_mir797	0	21	0.001	0.200666667	4.504314181	UP
novel_mir73	0	21	0.001	0.200666667	4.504314181	UP
novel_mir417	0	21	0.001	0.200666667	4.504314181	UP
novel_mir57	0	21	0.001	0.200666667	4.504314181	UP
novel_mir724	0	21	0.001	0.200666667	4.504314181	UP
mmu-miR-297b-3p	0	20	0.001	0.203333333	4.433924853	UP
novel_mir300	0	20	0.001	0.190666667	4.433924853	UP
novel_mir104	0	20	0.001	0.190666667	4.433924853	UP
novel_mir701	0	20	0.001	0.190666667	4.433924853	UP
novel_mir686	0	20	0.001	0.190666667	4.433924853	UP
mmu-miR-5114	13	66	0.150333333	0.663333333	1.455951159	UP
mmu-miR-182-3p	31	19	0.37	0.193333333	-1.594272039	DOWN
novel_mir397	10	0	0.107333333	0.001	-5.209931337	DOWN
novel_mir276	10	0	0.107333333	0.001	-5.209931337	DOWN
novel_mir737	10	0	0.107333333	0.001	-5.209931337	DOWN
novel_mir395	10	0	0.107333333	0.001	-5.209931337	DOWN
novel_mir279	10	0	0.107333333	0.001	-5.209931337	DOWN
novel_mir553	10	0	0.107333333	0.001	-5.209931337	DOWN
novel_mir733	10	0	0.107333333	0.001	-5.209931337	DOWN
novel_mir263	10	0	0.107333333	0.001	-5.209931337	DOWN

novel_mir718	23	11	0.244	0.104	-1.952133579	DOWN
novel_mir590	10	56	0.107333333	0.587	1.597423585	UP
novel_mir201	0	19	0.001	0.180666667	4.359924271	UP
novel_mir538	0	19	0.001	0.180666667	4.359924271	UP
novel_mir651	0	19	0.001	0.180666667	4.359924271	UP
novel_mir780	0	19	0.001	0.180666667	4.359924271	UP
novel_mir195	0	19	0.001	0.180666667	4.359924271	UP
novel_mir518	0	19	0.001	0.180666667	4.359924271	UP
novel_mir710	0	19	0.001	0.180666667	4.359924271	UP
novel_mir431	0	19	0.001	0.180666667	4.359924271	UP
novel_mir632	0	19	0.001	0.180666667	4.359924271	UP
novel_mir430	0	19	0.001	0.210666667	4.359924271	UP
novel_mir642	0	19	0.001	0.180666667	4.359924271	UP
mmu-let-7g-3p	8	48	0.100333333	0.496666667	1.696959259	UP
mmu-miR-770-5p	13	3	0.163333333	0.030333333	-3.003480459	DOWN
mmu-miR-6969-3p	13	3	0.163333333	0.030666667	-3.003480459	DOWN
mmu-miR-299a-5p	10	1	0.123333333	0.010666667	-4.209931337	DOWN
novel_mir181	40	31	0.427333333	0.297333333	-1.255735026	DOWN
novel_mir808	0	18	0.001	0.170666667	4.281921759	UP
novel_mir147	0	18	0.001	0.170666667	4.281921759	UP
novel_mir641	0	18	0.001	0.170666667	4.281921759	UP
novel_mir365	0	18	0.001	0.170666667	4.281921759	UP
novel_mir336	0	18	0.001	0.170666667	4.281921759	UP
mmu-miR-3966	22	88	0.234	0.89	1.111996758	UP
mmu-miR-3105-3p	26	98	0.300333333	0.976666667	1.026266884	UP
mmu-miR-3970	7	43	0.074	0.433333333	1.730906591	UP
novel_mir485	34	25	0.364	0.240666667	-1.331609893	DOWN
novel_mir183	0	17	0.001	0.164	4.199459599	UP
novel_mir470	0	17	0.001	0.164	4.199459599	UP
novel_mir203	0	17	0.001	0.164	4.199459599	UP
novel_mir761	0	17	0.001	0.164	4.199459599	UP
novel_mir812	0	17	0.001	0.164	4.199459599	UP
novel_mir171	0	17	0.001	0.164	4.199459599	UP
novel_mir764	0	17	0.001	0.164	4.199459599	UP
novel_mir244	10	52	0.107333333	0.503666667	1.490508381	UP
novel_mir551	14	63	0.150666667	0.613666667	1.281921759	UP
novel_mir302	0	16	0.001	0.154	4.111996758	UP
novel_mir227	0	16	0.001	0.154	4.111996758	UP

novel_mir592	0	16	0.001	0.177333333	4.111996758	UP
novel_mir255	0	16	0.001	0.154	4.111996758	UP
novel_mir594	0	16	0.001	0.154	4.111996758	UP
novel_mir461	0	16	0.001	0.154	4.111996758	UP
novel_mir402	0	16	0.001	0.154	4.111996758	UP
novel_mir208	0	16	0.001	0.154	4.111996758	UP
novel_mir74	0	16	0.001	0.154	4.111996758	UP
mmu-miR-1968-5p	48	44	0.59	0.443333333	-1.013534124	DOWN
mmu-miR-466k	17	8	0.220333333	0.077	-1.975466083	DOWN
mmu-miR-1306-3p	19	75	0.230333333	0.746666667	1.092887935	UP
novel_mir277	0	15	0.001	0.144	4.018887354	UP
novel_mir633	0	15	0.001	0.144	4.018887354	UP
novel_mir674	0	15	0.001	0.144	4.018887354	UP
novel_mir177	0	15	0.001	0.144	4.018887354	UP
novel_mir547	0	15	0.001	0.144	4.018887354	UP
novel_mir450	0	15	0.001	0.144	4.018887354	UP
mmu-miR-125b-2-	35	29	0.436666667	0.286666667	-1.159305264	DOWN
mmu-miR-341-5p	1	17	0.014	0.176666667	3.199459599	UP
mmu-miR-186-3p	2	21	0.023666667	0.206666667	2.504314181	UP
novel_mir693	0	14	0.001	0.134	3.91935168	UP
novel_mir561	0	14	0.001	0.134	3.91935168	UP
novel_mir160	0	14	0.001	0.134	3.91935168	UP
novel_mir437	0	14	0.001	0.134	3.91935168	UP
novel_mir58	0	14	0.001	0.134	3.91935168	UP
novel_mir322	0	14	0.001	0.134	3.91935168	UP
novel_mir156	0	14	0.001	0.134	3.91935168	UP
novel_mir268	0	14	0.001	0.134	3.91935168	UP
novel_mir320	0	14	0.001	0.134	3.91935168	UP
novel_mir389	0	14	0.001	0.134	3.91935168	UP
novel_mir342	0	14	0.001	0.134	3.91935168	UP
novel_mir120	0	14	0.001	0.134	3.91935168	UP
novel_mir250	0	14	0.001	0.134	3.91935168	UP
novel_mir235	0	14	0.001	0.134	3.91935168	UP
novel_mir616	0	14	0.001	0.134	3.91935168	UP
novel_mir529	35	30	0.417	0.287333333	-1.110395663	DOWN
mmu-miR-701-5p	16	64	0.186666667	0.633333333	1.111996758	UP
novel_mir131	11	50	0.117333333	0.477333333	1.296421329	UP

Supple Table 2: Differential miRNAs between IR and HRS+IR groups

miRNA id	Read count(IR)	Read count(HRS+IR)	Expression(IR)	Expression(HRS+IR)	log2Ratio(HRS+IR/IR)	Up down regulation
novel_mir506	740	10980	8.14	134.21	4.978910269	UP
mmu-miR-5126	6918	17453	71.11	209.0566667	2.42274942	UP
mmu-miR-199a-3p	21854	765	210.5633333	9.16	-3.74859251	DOWN
novel_mir676	757	8338	8.233333333	100.8133333	4.549037463	UP
novel_mir515	394	5926	4.150333333	61.76	4.998492388	UP
novel_mir607	344	5636	3.743333333	69.83333333	5.121892438	UP
novel_mir1	32918	31871	349.0333333	390.8033333	1.041068898	UP
mmu-miR-6538	1784	7216	17.96666667	85.01333333	3.103785019	UP
novel_mir119	1627	6888	16.89666667	87.26333333	3.169572188	UP
novel_mir755	24062	5398	233.72	68.05	-1.068558384	DOWN
novel_mir669	4141	79	39.70666667	1.023333333	-4.624281443	DOWN
novel_mir582	454	2081	4.563666667	24.42	3.284214059	UP
novel_mir5	467	1738	4.93	21.47	2.983634923	UP
novel_mir520	558	1747	5.716666667	21.69	2.734243877	UP
novel_mir508	149	1228	1.546666667	14.66666667	4.130627621	UP
mmu-let-7c-2-3p	4722	700	48.01333333	9.593666667	-1.666269918	DOWN
mmu-miR-342-3p	9281	2178	93.40666667	26.45	-1.00357501	DOWN
novel_mir785	44	586	0.443666667	6.556666667	4.823026532	UP
novel_mir496	289	819	3.12	10.22	2.590495255	UP
mmu-miR-296-5p	1749	1936	18.07666667	23.56666667	1.234249959	UP
mmu-miR-677-5p	2936	520	29.76333333	6.303333333	-1.409567144	DOWN
novel_mir473	28	364	0.300333333	4.276666667	4.788141014	UP
novel_mir259	826	29	7.917	0.397333333	-3.74431568	DOWN
novel_mir15	487	783	5.160333333	10.36333333	1.772791831	UP
novel_mir468	175	471	1.8	5.85	2.516073434	UP
mmu-miR-574-5p	2767	602	27.70666667	7.363333333	-1.112785956	DOWN
novel_mir465	55	256	0.597	3.173333333	3.306341583	UP
novel_mir6	1867	377	18.94666667	4.803333333	-1.220384203	DOWN
mmu-let-7a-1-3p	1004	1017	10.25666667	11.67	1.106261706	UP
novel_mir495	1872	392	19.73	4.516666667	-1.167953579	DOWN
novel_mir297	696	66	6.663666667	0.840333333	-2.31084808	DOWN
novel_mir585	338	504	3.463333333	6.186666667	1.664101783	UP
novel_mir8	494	626	5.066666667	8.046666667	1.429352911	UP
novel_mir491	41	211	0.457333333	2.667	3.45124848	UP
mmu-miR-2137	473	583	4.933333333	6.32	1.389356996	UP
mmu-miR-3470b	268	408	2.676666667	5.023333333	1.694037448	UP

mmu-miR-3470a	289	421	2.896666667	5.14	1.630452037	UP
mmu-miR-532-3p	45	180	0.466666667	2.263333333	3.087701296	UP
novel_mir525	0	173	0.001	2.083333333	9.522329524	UP
novel_mir14	900	148	9.536666667	1.893333333	-1.516626529	DOWN
novel_mir610	28	143	0.267333333	1.857	3.440217711	UP
novel_mir240	96	214	1.003333333	2.543333333	2.244205782	UP
mmu-miR-714	98	212	1.006666667	2.58	2.200911907	UP
mmu-miR-5121	808	142	8.053333333	1.8	-1.420763067	DOWN
novel_mir19	456	49	4.433333333	0.674	-2.130478874	DOWN
novel_mir228	228	0	2.263666667	0.001	-7.745188718	DOWN
novel_mir22	440	51	4.197333333	0.700666667	-2.021233075	DOWN
novel_mir471	283	349	2.95	4.326666667	1.390126279	UP
novel_mir72	57	153	0.6	1.803333333	2.512199125	UP
novel_mir689	199	0	1.897333333	0.001	-7.548923324	DOWN
mmu-miR-338-3p	45	132	0.493333333	1.62	2.640242319	UP
novel_mir656	183	0	1.744	0.001	-7.427998542	DOWN
novel_mir87	362	40	4.010333333	0.523666667	-2.090216496	DOWN
novel_mir448	0	91	0.001	1.090333333	8.595495936	UP
novel_mir657	220	277	2.416666667	3.396666667	1.420083749	UP
novel_mir722	111	183	1.16	2.163333333	1.808985268	UP
novel_mir354	83	157	0.867	1.993333333	2.007282614	UP
novel_mir659	21	90	0.200666667	1.113333333	3.18723697	UP
novel_mir18	136	194	1.4	2.52	1.600151297	UP
novel_mir13	53	118	0.507333333	1.553666667	2.242423891	UP
novel_mir163	11	70	0.124	0.873666667	3.757552694	UP
mmu-miR-3065-5p	423	70	4.243333333	0.88	-1.50752954	DOWN
novel_mir89	0	66	0.001	0.777333333	8.132095415	UP
novel_mir680	46	101	0.483666667	1.33	2.222350823	UP
novel_mir452	100	0	0.954	0.001	-6.556154894	DOWN
novel_mir141	262	259	2.676666667	3.113333333	1.071086582	UP
novel_mir619	96	0	0.930333333	0.001	-6.497261205	DOWN
novel_mir24	338	59	3.25	0.810666667	-1.430535091	DOWN
novel_mir600	94	0	0.897333333	0.001	-6.466887556	DOWN
novel_mir202	0	47	0.001	0.573666667	7.642290148	UP
mmu-miR-6240	501	111	4.993333333	1.306666667	-1.086549631	DOWN
novel_mir729	85	0	0.810666667	0.001	-6.32168964	DOWN
novel_mir766	82	0	0.784	0.001	-6.269850709	DOWN
mmu-miR-18b-5p	217	215	2.193333333	2.493333333	1.074342913	UP

novel_mir38	81	0	0.774	0.001	-6.252148707	DOWN
novel_mir580	80	0	0.773666667	0.001	-6.234226799	DOWN
novel_mir599	80	0	0.777	0.001	-6.234226799	DOWN
mmu-miR-6236	7	44	0.077	0.540333333	3.739777993	UP
novel_mir70	10	46	0.097333333	0.543333333	3.289335157	UP
novel_mir124	0	36	0.001	0.494	7.257626298	UP
novel_mir155	161	19	1.537333333	0.260666667	-1.995288069	DOWN
novel_mir386	126	11	1.200666667	0.130666667	-2.430147009	DOWN
novel_mir802	0	35	0.001	0.387	7.216984313	UP
novel_mir587	69	0	0.667	0.001	-6.020823161	DOWN
novel_mir784	68	0	0.657	0.001	-5.999761545	DOWN
novel_mir783	230	40	2.227	0.550666667	-1.43586066	DOWN
novel_mir511	63	0	0.600666667	0.001	-5.889578627	DOWN
novel_mir551	63	0	0.613666667	0.001	-5.889578627	DOWN
novel_mir588	0	30	0.001	0.410666667	6.994591892	UP
novel_mir126	60	0	0.590333333	0.001	-5.8191893	DOWN
novel_mir571	60	0	0.574	0.001	-5.8191893	DOWN
novel_mir410	60	0	0.574	0.001	-5.8191893	DOWN
novel_mir700	0	29	0.001	0.340666667	6.945682291	UP
novel_mir482	59	0	0.600666667	0.001	-5.794941753	DOWN
novel_mir444	57	0	0.544	0.001	-5.745188718	DOWN
novel_mir278	57	0	0.544	0.001	-5.745188718	DOWN
novel_mir611	12	40	0.114	0.550666667	2.82466689	UP
novel_mir590	56	0	0.587	0.001	-5.719653626	DOWN
novel_mir514	200	35	1.910333333	0.480666667	-1.426871877	DOWN
novel_mir541	54	0	0.546666667	0.001	-5.667186206	DOWN
novel_mir626	54	0	0.514	0.001	-5.667186206	DOWN
novel_mir244	52	0	0.503666667	0.001	-5.612738422	DOWN
novel_mir801	0	25	0.001	0.344	6.731557486	UP
novel_mir405	50	0	0.490333333	0.001	-5.556154894	DOWN
novel_mir190	50	0	0.477333333	0.001	-5.556154894	DOWN
novel_mir53	49	0	0.467333333	0.001	-5.527008548	DOWN
mmu-miR-351-5p	282	64	2.866666667	0.766666667	-1.051850056	DOWN
novel_mir359	0	23	0.001	0.317333333	6.611263252	UP
novel_mir114	48	0	0.457333333	0.001	-5.497261205	DOWN
novel_mir39	121	16	1.154	0.220666667	-1.831161941	DOWN
novel_mir180	134	20	1.283666667	0.274	-1.656459799	DOWN
novel_mir59	46	0	0.440666667	0.001	-5.43586066	DOWN

novel_mir576	45	0	0.480333333	0.001	-5.4041518	DOWN
novel_mir213	106	109	1.046666667	1.497333333	1.127965166	UP
novel_mir527	0	21	0.001	0.287333333	6.480018719	UP
novel_mir182	44	0	0.420666667	0.001	-5.371730323	DOWN
mmu-miR-3084-3p	18	40	0.18	0.476666667	2.23970439	UP
novel_mir267	43	0	0.410666667	0.001	-5.338563459	DOWN
novel_mir441	43	0	0.410666667	0.001	-5.338563459	DOWN
novel_mir258	43	0	0.410666667	0.001	-5.338563459	DOWN
mmu-miR-322-3p	235	52	2.363333333	0.636666667	-1.088375932	DOWN
novel_mir570	0	20	0.001	0.237333333	6.409629391	UP
novel_mir337	0	20	0.001	0.253666667	6.409629391	UP
novel_mir34	150	26	1.430666667	0.357333333	-1.440677676	DOWN
novel_mir149	42	0	0.400666667	0.001	-5.304616127	DOWN
novel_mir231	49	65	0.477	0.870333333	1.495359265	UP
novel_mir504	41	0	0.390666667	0.001	-5.269850709	DOWN
novel_mir138	0	19	0.001	0.260666667	6.33562881	UP
novel_mir768	40	0	0.380666667	0.001	-5.234226799	DOWN
novel_mir523	0	18	0.001	0.247333333	6.257626298	UP
novel_mir770	38	0	0.364	0.001	-5.160226217	DOWN
novel_mir321	38	0	0.364	0.001	-5.160226217	DOWN
novel_mir117	37	0	0.354	0.001	-5.12175207	DOWN
novel_mir608	37	0	0.367	0.001	-5.12175207	DOWN
mmu-miR-17-3p	221	51	2.223333333	0.61	-1.027775921	DOWN
novel_mir645	0	17	0.001	0.234	6.175164137	UP
novel_mir746	0	17	0.001	0.234	6.175164137	UP
novel_mir579	117	19	1.117333333	0.260666667	-1.53473591	DOWN
mmu-miR-669c-5p	144	27	1.463333333	0.340333333	-1.327336203	DOWN
mmu-miR-331-3p	34	49	0.37	0.613333333	1.614948299	UP
novel_mir252	35	0	0.360333333	0.001	-5.041581721	DOWN
novel_mir101	35	0	0.334	0.001	-5.041581721	DOWN
novel_mir652	35	0	0.334	0.001	-5.041581721	DOWN
novel_mir557	35	0	0.356666667	0.001	-5.041581721	DOWN
novel_mir403	35	0	0.334	0.001	-5.041581721	DOWN
novel_mir457	0	16	0.001	0.187333333	6.087701296	UP
novel_mir100	0	16	0.001	0.220666667	6.087701296	UP
novel_mir366	0	16	0.001	0.220666667	6.087701296	UP
novel_mir130	0	16	0.001	0.220666667	6.087701296	UP
novel_mir400	34	0	0.324	0.001	-4.999761545	DOWN

novel_mir148	34	0	0.324	0.001	-4.999761545	DOWN
novel_mir49	34	0	0.324	0.001	-4.999761545	DOWN
novel_mir218	79	10	0.860333333	0.137333333	-1.894151357	DOWN
mmu-miR-3068-3p	6	22	0.063333333	0.26	2.962170414	UP
novel_mir198	33	0	0.314	0.001	-4.956692823	DOWN
novel_mir60	33	0	0.314	0.001	-4.956692823	DOWN
novel_mir61	33	0	0.314	0.001	-4.956692823	DOWN
novel_mir346	33	0	0.314	0.001	-4.956692823	DOWN
novel_mir562	33	0	0.314	0.001	-4.956692823	DOWN
novel_mir107	0	15	0.001	0.207333333	5.994591892	UP
novel_mir666	31	0	0.314	0.001	-4.866495014	DOWN
novel_mir476	31	0	0.297333333	0.001	-4.866495014	DOWN
novel_mir52	31	0	0.297333333	0.001	-4.866495014	DOWN
mmu-miR-23b-5p	182	42	1.803333333	0.53	-1.027775921	DOWN
novel_mir762	0	14	0.001	0.194	5.895056218	UP
novel_mir113	0	14	0.001	0.194	5.895056218	UP
novel_mir800	0	14	0.001	0.194	5.895056218	UP
novel_mir731	30	0	0.287333333	0.001	-4.8191893	DOWN
novel_mir715	30	0	0.287333333	0.001	-4.8191893	DOWN
novel_mir782	30	0	0.287333333	0.001	-4.8191893	DOWN
novel_mir568	30	0	0.287333333	0.001	-4.8191893	DOWN
novel_mir167	86	13	0.833666667	0.177333333	-1.63812374	DOWN
novel_mir421	29	0	0.277333333	0.001	-4.770279699	DOWN
novel_mir95	29	0	0.297	0.001	-4.770279699	DOWN
novel_mir629	29	0	0.277333333	0.001	-4.770279699	DOWN
novel_mir230	29	0	0.277333333	0.001	-4.770279699	DOWN
novel_mir188	29	0	0.277333333	0.001	-4.770279699	DOWN
novel_mir477	29	0	0.324	0.001	-4.770279699	DOWN
novel_mir425	29	0	0.277333333	0.001	-4.770279699	DOWN
novel_mir170	60	64	0.596666667	0.807	1.1808107	UP
novel_mir542	0	13	0.001	0.177333333	5.788141014	UP
novel_mir153	0	13	0.001	0.177333333	5.788141014	UP
novel_mir733	0	13	0.001	0.177333333	5.788141014	UP
novel_mir77	0	13	0.001	0.154	5.788141014	UP
novel_mir251	0	13	0.001	0.154	5.788141014	UP
novel_mir165	28	0	0.267333333	0.001	-4.719653626	DOWN
novel_mir398	28	0	0.314	0.001	-4.719653626	DOWN
novel_mir672	28	0	0.267333333	0.001	-4.719653626	DOWN

novel_mir671	28	0	0.267333333	0.001	-4.719653626	DOWN
novel_mir544	27	0	0.257333333	0.001	-4.667186206	DOWN
novel_mir335	27	0	0.257333333	0.001	-4.667186206	DOWN
novel_mir493	27	0	0.263666667	0.001	-4.667186206	DOWN
novel_mir490	27	0	0.257333333	0.001	-4.667186206	DOWN
novel_mir222	27	0	0.257333333	0.001	-4.667186206	DOWN
novel_mir123	27	0	0.257333333	0.001	-4.667186206	DOWN
novel_mir613	27	0	0.257333333	0.001	-4.667186206	DOWN
novel_mir563	27	0	0.257333333	0.001	-4.667186206	DOWN
novel_mir376	27	0	0.257333333	0.001	-4.667186206	DOWN
novel_mir419	0	12	0.001	0.164	5.672663797	UP
novel_mir566	0	12	0.001	0.164	5.672663797	UP
novel_mir581	0	12	0.001	0.164	5.672663797	UP
novel_mir618	0	12	0.001	0.164	5.672663797	UP
novel_mir685	0	12	0.001	0.164	5.672663797	UP
novel_mir212	0	12	0.001	0.164	5.672663797	UP
novel_mir732	0	12	0.001	0.140666667	5.672663797	UP
novel_mir573	0	12	0.001	0.164	5.672663797	UP
novel_mir162	0	12	0.001	0.164	5.672663797	UP
novel_mir772	0	12	0.001	0.164	5.672663797	UP
novel_mir344	0	12	0.001	0.140666667	5.672663797	UP
novel_mir323	26	0	0.247333333	0.001	-4.612738422	DOWN
novel_mir429	26	0	0.247333333	0.001	-4.612738422	DOWN
novel_mir728	26	0	0.247333333	0.001	-4.612738422	DOWN
novel_mir443	26	0	0.267	0.001	-4.612738422	DOWN
novel_mir237	76	12	0.753333333	0.164	-1.575263717	DOWN
novel_mir159	25	0	0.240666667	0.001	-4.556154894	DOWN
novel_mir485	25	0	0.240666667	0.001	-4.556154894	DOWN
novel_mir79	25	0	0.240666667	0.001	-4.556154894	DOWN
novel_mir705	25	0	0.240666667	0.001	-4.556154894	DOWN
novel_mir91	25	0	0.240666667	0.001	-4.556154894	DOWN
novel_mir754	0	11	0.001	0.150666667	5.547132915	UP
novel_mir454	0	11	0.001	0.150666667	5.547132915	UP
novel_mir614	0	11	0.001	0.130666667	5.547132915	UP
novel_mir392	0	11	0.001	0.130666667	5.547132915	UP
novel_mir727	0	11	0.001	0.150666667	5.547132915	UP
novel_mir795	16	27	0.154	0.370666667	1.842588798	UP
novel_mir303	24	0	0.230666667	0.001	-4.497261205	DOWN

novel_mir110	24	0	0.230666667	0.001	-4.497261205	DOWN
novel_mir776	24	0	0.230666667	0.001	-4.497261205	DOWN
mmu-miR-3071-5p	15	26	0.157	0.31	1.881250419	UP
novel_mir574	65	63	0.647	0.864	1.042613407	UP
novel_mir99	30	38	0.287333333	0.520666667	1.428738214	UP
novel_mir638	73	12	0.737	0.140666667	-1.517160762	DOWN
novel_mir219	23	0	0.220666667	0.001	-4.43586066	DOWN
novel_mir699	23	0	0.220666667	0.001	-4.43586066	DOWN
novel_mir385	23	0	0.220666667	0.001	-4.43586066	DOWN
novel_mir549	0	10	0.001	0.117333333	5.409629391	UP
novel_mir233	0	10	0.001	0.117333333	5.409629391	UP
novel_mir445	0	10	0.001	0.117333333	5.409629391	UP
novel_mir30	0	10	0.001	0.137333333	5.409629391	UP
novel_mir753	0	10	0.001	0.137333333	5.409629391	UP
novel_mir439	0	10	0.001	0.137333333	5.409629391	UP
novel_mir735	0	10	0.001	0.137333333	5.409629391	UP
novel_mir94	0	10	0.001	0.137333333	5.409629391	UP
novel_mir115	22	0	0.210666667	0.001	-4.371730323	DOWN
novel_mir64	22	0	0.210666667	0.001	-4.371730323	DOWN
novel_mir150	22	0	0.210666667	0.001	-4.371730323	DOWN
novel_mir507	22	0	0.210666667	0.001	-4.371730323	DOWN
novel_mir76	22	0	0.210666667	0.001	-4.371730323	DOWN
novel_mir781	22	0	0.210666667	0.001	-4.371730323	DOWN
novel_mir78	28	35	0.277	0.437	1.409629391	UP
novel_mir628	21	0	0.200666667	0.001	-4.304616127	DOWN
novel_mir248	21	0	0.200666667	0.001	-4.304616127	DOWN
novel_mir84	21	0	0.214	0.001	-4.304616127	DOWN
novel_mir797	21	0	0.200666667	0.001	-4.304616127	DOWN
novel_mir73	21	0	0.200666667	0.001	-4.304616127	DOWN
novel_mir417	21	0	0.200666667	0.001	-4.304616127	DOWN
novel_mir57	21	0	0.200666667	0.001	-4.304616127	DOWN
novel_mir528	21	0	0.200666667	0.001	-4.304616127	DOWN
mmu-miR-935	111	25	1.1	0.316666667	-1.06285838	DOWN
novel_mir46	47	48	0.447333333	0.620333333	1.118074945	UP
novel_mir35	101	22	0.977	0.304	-1.111078568	DOWN
novel_mir505	113	26	1.090333333	0.330333333	-1.032037948	DOWN
novel_mir102	61	10	0.593666667	0.137333333	-1.521107947	DOWN
novel_mir467	20	0	0.190666667	0.001	-4.234226799	DOWN

novel_mir54	20	0	0.207	0.001	-4.234226799	DOWN
novel_mir261	20	0	0.190666667	0.001	-4.234226799	DOWN
novel_mir104	20	0	0.190666667	0.001	-4.234226799	DOWN
novel_mir176	20	0	0.190666667	0.001	-4.234226799	DOWN
novel_mir701	20	0	0.190666667	0.001	-4.234226799	DOWN
novel_mir686	20	0	0.190666667	0.001	-4.234226799	DOWN
novel_mir201	19	0	0.180666667	0.001	-4.160226217	DOWN
novel_mir538	19	0	0.180666667	0.001	-4.160226217	DOWN
novel_mir651	19	0	0.180666667	0.001	-4.160226217	DOWN
novel_mir780	19	0	0.180666667	0.001	-4.160226217	DOWN
novel_mir195	19	0	0.180666667	0.001	-4.160226217	DOWN
novel_mir518	19	0	0.180666667	0.001	-4.160226217	DOWN
novel_mir710	19	0	0.180666667	0.001	-4.160226217	DOWN
novel_mir431	19	0	0.180666667	0.001	-4.160226217	DOWN
novel_mir632	19	0	0.180666667	0.001	-4.160226217	DOWN
novel_mir430	19	0	0.210666667	0.001	-4.160226217	DOWN
novel_mir642	19	0	0.180666667	0.001	-4.160226217	DOWN
novel_mir409	14	22	0.134	0.304	1.739777993	UP
mmu-miR-34b-3p	98	22	0.993333333	0.273333333	-1.067576929	DOWN
mmu-miR-3473d	58	10	0.573333333	0.126666667	-1.448351604	DOWN
novel_mir641	18	0	0.170666667	0.001	-4.082223705	DOWN
novel_mir365	18	0	0.170666667	0.001	-4.082223705	DOWN
novel_mir336	18	0	0.170666667	0.001	-4.082223705	DOWN
mmu-miR-669a-3p	8	16	0.090666667	0.196666667	2.087701296	UP
mmu-miR-190a-3p	74	15	0.74	0.183333333	-1.214861474	DOWN
mmu-miR-877-5p	39	5	0.396666667	0.06	-1.875772828	DOWN
novel_mir805	28	32	0.267333333	0.440666667	1.280346374	UP
novel_mir223	26	30	0.290666667	0.410666667	1.294152174	UP
novel_mir183	17	0	0.164	0.001	-3.999761545	DOWN
novel_mir203	17	0	0.164	0.001	-3.999761545	DOWN
novel_mir761	17	0	0.164	0.001	-3.999761545	DOWN
novel_mir812	17	0	0.164	0.001	-3.999761545	DOWN
novel_mir171	17	0	0.164	0.001	-3.999761545	DOWN
novel_mir283	17	0	0.164	0.001	-3.999761545	DOWN
novel_mir798	17	0	0.164	0.001	-3.999761545	DOWN
novel_mir764	17	0	0.164	0.001	-3.999761545	DOWN
novel_mir179	75	16	0.717333333	0.187333333	-1.141117394	DOWN
